# Association mapping in *Salix viminalis* L. (Salicaceae) – identification of candidate genes associated with growth and phenology

**DOI:** 10.1111/gcbb.12280

**Published:** 2015-07-29

**Authors:** Henrik R. Hallingbäck, Johan Fogelqvist, Stephen J. Powers, Juan Turrion‐Gomez, Rachel Rossiter, Joanna Amey, Tom Martin, Martin Weih, Niclas Gyllenstrand, Angela Karp, Ulf Lagercrantz, Steven J. Hanley, Sofia Berlin, Ann‐Christin Rönnberg‐Wästljung

**Affiliations:** ^1^ Department of Plant BiologyUppsala BioCenterSwedish University of Agricultural Sciences and Linnean Center for Plant BiologyP.O. Box 7043750 07UppsalaSweden; ^2^ Computational and Systems Biology DepartmentRothamsted ResearchHarpendenHertsAL5 2JQUK; ^3^ AgroEcology DepartmentRothamsted ResearchHarpendenHertsAL5 2JQUK; ^4^ Department of Crop Production EcologySwedish University of Agricultural Sciences and Linnean Center for Plant BiologyP.O. Box 7043750 07UppsalaSweden; ^5^ Department of Plant Ecology and EvolutionEvolutionary Biology CentreUppsala University752 36UppsalaSweden

**Keywords:** adaptation, association mapping, candidate gene, growth, marker‐assisted selection, phenology, *Salix*, short‐rotation coppice, willow

## Abstract

Willow species (*Salix*) are important as short‐rotation biomass crops for bioenergy, which creates a demand for faster genetic improvement and breeding through deployment of molecular marker‐assisted selection (MAS). To find markers associated with important adaptive traits, such as growth and phenology, for use in MAS, we genetically dissected the trait variation of a *Salix viminalis* (L.) population of 323 accessions. The accessions were sampled throughout northern Europe and were established at two field sites in Pustnäs, Sweden, and at Woburn, UK, offering the opportunity to assess the impact of genotype‐by‐environment interactions (G × E) on trait–marker associations. Field measurements were recorded for growth and phenology traits. The accessions were genotyped using 1536 SNP markers developed from phenology candidate genes and from genes previously observed to be differentially expressed in contrasting environments. Association mapping between 1233 of these SNPs and the measured traits was performed taking into account population structure and threshold selection bias. At a false discovery rate (FDR) of 0.2, 29 SNPs were associated with bud burst, leaf senescence, number of shoots or shoot diameter. The percentage of accession variation ($R_{\mathrm{adj}}^{2}$) explained by these associations ranged from 0.3% to 4.4%, suggesting that the studied traits are controlled by many loci of limited individual impact. Despite this, a SNP in the *EARLY FLOWERING 3* gene was repeatedly associated (FDR < 0.2) with bud burst. The rare homozygous genotype exhibited 0.4–1.0 lower bud burst scores than the other genotype classes on a five‐grade scale. Consequently, this marker could be promising for use in MAS and the gene deserves further study. Otherwise, associations were less consistent across sites, likely due to their small $R_{\mathrm{adj}}^{2}$ estimates and to considerable G × E interactions indicated by multivariate association analyses and modest trait accession correlations across sites (0.32–0.61).

## Introduction

In the cultivation of trees and shrubs for biomass production, fast and stable growth is a highly desirable trait. To achieve this, the plant material needs to be genetically adapted to the environment at the site where it is cultivated. Drought tolerance, cold tolerance, nutrient use efficiency and resistance to pathogens are all examples of adaptive traits. However, phenological characters, such as the timing of bud set, growth cessation and leaf senescence in the autumn, and bud burst and growth initiation in spring, are considered to be especially important as these responses determine the length of the growing season (Weih, 2009; Olsen, 2010; Paul *et al*., 2014). The timing of phenological growth transitions is highly influenced by a combination of temperature and photoperiodic cues (Cannell, 1990; Fracheboud *et al*., 2009). The plant integrates these onto developmental switches in a delicate trade‐off between the competitive advantage of early and longer growth versus possible damage from low/freezing temperatures in spring and autumn (Savage & Cavender‐Bares, 2013). Concerns have been raised that long‐lived perennials will become locally maladapted as a result of predicted global climate changes (Alberto *et al*., 2013). However, given that phenology traits usually are highly heritable, readaptation of the phenology is possible, and selective breeding programmes with the aim of improving or adjusting these traits have been established for many perennial biomass crops, for example *Panicum, Miscanthus, Populus* and *Salix* spp. (Karp & Shield, 2008).

In addition to traditional selection and breeding methods, the development of DNA‐based technologies has opened up the opportunity to develop molecular markers that are associated with traits of interest. Breeders can now select suitable genotypes in the breeding process and for propagation based on marker genotype information (marker‐assisted selection MAS, see Thavamanikumar *et al*., 2013) rather than on phenotypic evaluation alone. MAS could be particularly beneficial in perennials with long yield cycles and generation times, such as trees, as individuals with desired characteristics can be identified already at the seedling stage, thereby reducing the need for time‐consuming and costly field testing (Harfouche *et al*., 2012). The detection of associations also increases the general knowledge on the nature of the genetic control of economically and ecologically relevant traits. For the development of such selection tools, a dissection of the relevant traits by, for example, quantitative trait locus (QTL) linkage mapping or by association mapping is first required (Savolainen *et al*., 2013; Hanley & Karp, 2014).

Association mapping is a method for detecting and characterizing the effects of individual genetic loci on complex traits (e.g. Lander & Schork, 1994; Pritchard *et al*., 2000b). In plant populations of largely unrelated individuals, the correlation between loci (linkage disequilibrium, LD) is expected to be very low due to the extensive number of recombination events that have occurred during the many generations since the latest common ancestor (Remington *et al*., 2001; Thornsberry *et al*., 2001). Markers observed to be associated with interesting traits are therefore likely to be situated very close to the causal locus (e.g. the gene actually affecting the trait). Due to the tightness of this linkage, trait‐associated markers identified by association mapping are more likely to be applicable in MAS in a wider set of pedigrees and populations than is usually found for markers identified by linkage mapping (Neale & Savolainen, 2004).

Association mapping analyses of growth and phenology traits have previously been performed with varying success in a number of tree species, for example *Populus tremula* (Ingvarsson *et al*., 2008), *Pseudotsuga menziesii* var *menziesii* (Eckert *et al*., 2009), *Picea sitchensis* (Bong.) Carr (Holliday *et al*., 2010), *Populus balsamifera* (L.) (Olson *et al*., 2013), *Pinus taeda* (L.) (Chhatre *et al*., 2013), *Picea marinara* (Mill.) (Prunier *et al*., 2013) and *Populus trichocarpa* (Torr. & Gray) (Evans *et al*., 2014; McKown *et al*., 2014). A versatile feature of association mapping is that, unlike linkage mapping, it is not dependent on the construction of a dense linkage map. The ambition and genotyping efforts spent on an association mapping study can therefore range from using relatively few markers carefully chosen in genes previously known to be connected to the studied traits (candidate gene approach), to the use of an extensive number of markers, densely and randomly distributed across the entire genome (genomewide approach). The candidate gene approach has been the most popular in previous studies because of the possibility to use the existing genomic resources accumulated for model species (e.g. *Arabidopsis thaliana* or *Populus trichocarpa*) to obtain a limited number of potentially important single nucleotide polymorphism (SNP) markers in target genes. In the nonmodel tree species, the lack of sufficient resources has, hitherto, made large‐scale genomewide association mapping efforts unfeasible.

In addition to identifying markers associated with adaptive traits *per se*, it is also important to determine the extent to which these associations are dependent on the local environment (sometimes termed *conditional neutrality*, Savolainen *et al*., 2013) and thus to what extent association mapping results can be generalized. In the light of adaptation to global climate change, focus on this aspect of genetic dissection has gained some urgency. Also with respect to genetic improvement and breeding, it is preferable that the effects linked to markers proposed for MAS are consistent in the different environments in which the plant material will need to be grown. However, among association mapping studies in trees, only a limited number of studies have investigated this aspect (Olson *et al*., 2013; Prunier *et al*., 2013; Evans *et al*., 2014).

The willow species *Salix viminalis* L. (common osier) is a pioneer outcrossing shrub or small tree that is frequently grown as a short‐rotation coppice (SRC) for bioenergy purposes in cool‐temperate regions (Karp & Shield, 2008; Kuzovkina *et al*., 2008). Due to its fast rate of maturation compared to other forest trees, willow may also be used as a model species for studies in genetics and breeding strategy (Hanley & Karp, 2014). Previous quantitative genetic studies in willows have revealed that ample genetic variation exists for growth and phenology, as well as many other traits (e.g. Rönnberg‐Wästljung & Gullberg, 1999), and a number of linkage mapping studies have identified genomic regions specifically associated with growth and phenology traits (e.g. Tsarouhas *et al*., 2002, 2003; Ghelardini *et al*., 2014). Many of these genomic regions are not well resolved but, to the knowledge of the authors, association mapping approaches, which could lead to tighter resolution of trait–marker associations, have not yet been conducted for any willow species. Candidate gene approaches are currently favoured due to the increased opportunity to develop numerous candidate SNPs based on previous linkage mapping or gene expression studies. SNP development is further facilitated by the close relationship between *Salix* spp. and *P. trichocarpa*, the latter of these having a published and well‐annotated genome sequence available (Tuskan *et al*., 2006).

The long‐term goal, to which this study will contribute, is to assess the potential for marker‐assisted selection of adaptive traits such as biomass growth and characteristics of spring and autumn phenology in *S. viminalis*. The particular objective of this study was to determine the extent to which the phenotypic variation for these traits was associated with allelic variation in a set of candidate gene‐derived SNPs. A secondary objective was to evaluate whether any marker–trait associations for the adaptive traits identified here were stable across contrasting environments. These objectives were pursued by applying an association mapping analysis on a willow population planted in two field trials in Sweden and the UK.

## Material and methods

### Material sampling and field trials

The association mapping population comprised a diverse collection of supposedly unrelated *S. viminalis* accessions that originated from the UK, Sweden, Belgium, Germany and western Russia (Berlin *et al*., 2014), augmented by recently collected material from *natural willow stands in* the Czech Republic (Trybush *et al*., 2012). Samples originated from latitudes between 48.1°N and 62.4°N and longitudes from 4.8°W to 104.3°E. In spring 2009, field experiments were established at two different sites: Pustnäs, south of Uppsala, Sweden (59.80°N, 17.67°E, 25 m AOD), and Woburn, UK (52.01°N, 0.59°W, 95 m AOD). A total of 387 accessions in Pustnäs and 397 at Woburn were planted with 20‐cm cuttings at a density of 15 000 *ha* ^−1^. Each accession was represented by six clonal replicates per experiment arranged in a randomized complete block design. The spacing was 130 cm between rows and 50 cm between plants within rows. To avoid border effects, two rows of the accession 78183 were planted outside the experimental plants. No fertilization was made during the first growing season, but during 2010 and 2011, fertilization (N P K; 21‐4‐7) corresponding to 80 kg N *ha* ^−1^ was applied. The plants were cut back in winter 2009/2010. Further details about the germplasm collection are found in Berlin *et al*. (2014).

### Phenotypic measurements

Leaf bud burst was assessed on each individual plant in both field experiments during spring 2010 and 2011, and in Pustnäs only in 2013, using a scale between 1 and 5 where 1 equals no sign of bud growth and 5 equals the most developed bud burst stage, with one or more leaves growing perpendicular to the shoot axis (Weih, 2009). In Pustnäs, the bud burst assessments were repeated every second day during a 2‐week period in late April and early May to find the timepoint for each year where the most variation in bud burst was observed (5 May 2010, 15 April 2011 and 2 May 2013, respectively). These timepoints were chosen as traits for further analyses (BB10, BB11 and BB13, Table 1). In Woburn, one timepoint each year was chosen (25 March 2010 and 4 March 2011). Growth cessation, defined by shoot apex abscission, was assessed in Pustnäs in autumn 2010 (GC10) on the highest shoot of each plant. From the end of August onwards, the plants were scored for growth cessation once or twice weekly. Plants that had reached growth cessation by the first day of measurement were given a value of 1, and the rest of the plants were given values corresponding to the number of days for the plant to reach growth cessation counted from the first day of measurements. Leaf senescence and abscission were visually assessed in Pustnäs on 4 November and 5 November 2010 (LS10), on 31 October 2011 (LS11) and in Woburn on 25 October 2010 (LS10) according to a leaf senescence index (LSI) from 0 to 4 with 0 = no leaves left on the plant (100% abscission) and 4 = more than 80% green leaves (∼10% abscission) (Ghelardini *et al*., 2014). During winter 2010/2011, two growth traits closely related to total biomass (Nordh & Verwijst, 2004) were assessed at both sites. The number of shoots (Nsh11) was counted, and the diameters on all shoots above 55 cm from the ground were measured. Mean diameter (MeanD11), maximum diameter (MaxD11) and summed shoot diameter (SumD11) were calculated and used for analysis.

**Table 1:** Summary statistics for the traits: abbreviations, measurement units, number of plants measured, overall means (per plant) and individual standard deviations (SD) for Pustnäs and Woburn

Trait	Abbr.	Unit	Pustnäs			Woburn		
			No. obs.	Mean	SD	No. obs.	Mean	SD
Spring phenology traits								
Bud burst stage 2010	BB10	Score	2238	3.30	0.57	2392	2.00	0.38
Bud burst stage 2011	BB11	Score	2251	2.44	0.72	2369	1.74	0.92
Bud burst stage 2013	BB13	Score	1502	2.93	0.70	–	–	–
Autumn phenology traits								
Days to GC 2010	GC10	No.	2235	23.05	11.73	–	–	–
Leaf senescence 2010	LS10	Score	2255	2.29	0.76	2392	2.56	0.51
Leaf senescence 2011	LS11	Score	2247	1.59	0.71	–	–	–
Biomass growth traits								
No. of shoots 2011	Nsh11	No.	2258	9.44	5.55	2371	13.08	6.04
Mean shoot diameter 2011	MeanD11	mm	2245	7.33	1.58	2370	7.58	1.56
Max shoot diameter 2011	MaxD11	mm	2245	10.33	2.19	2370	11.58	2.40
Summed shoot diameter 2011	SumD11	mm	2245	58.64	36.14	2370	98.89	50.06

GC, Growth cessation.

### Selection of candidate genes

**Figure 1:**
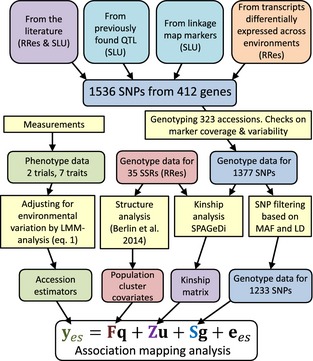
Schematic overview of the structure of the candidate SNP development and preparatory work for association analysis.

Candidate genes used in this study (Fig. 1) were selected based on (i) evidence in the literature suggesting they were relevant to phenology or plant drought stress; (ii) proximity to previously detected QTL for phenology (Ghelardini *et al*., 2014); and (iii) candidate gene lists generated previously in a transcriptome comparison of two willow genotypes growing on both productive and less‐productive field sites at Rothamsted's Woburn farm (unpublished data). The third group of genes was included under the supposition that genes expressed differentially in contrasting environments that are challenging to growth are likely to be of functional importance and as such, potentially interesting candidates for growth traits in an association study using different field testing sites. Three *MAX* gene homologues (*SxMAX1, SxMAX2* and *SxMAX4*) were also included to test for possible associations with plant architecture, following previous work indicating that *MAX* genes may influence growth traits in willows (Salmon *et al*., 2014). Full details are provided in Table S1.

### SNP discovery and genotyping

Two closely related SNP discovery protocols were used by the laboratories of SLU (Swedish University of Agricultural Sciences) and RRes (Rothamsted Research). At SLU, candidate gene homologues were first identified in the *P. trichocarpa* genome sequence (phytozome.net) and primers were designed for amplification of the willow orthologue. Primer3 software v1.1.3 (Rozen & Skaletsky, 2000) was used for primer design. Due to the largely duplicated nature of *Salicaceae* genomes, care was taken to avoid amplification of paralogous loci by ensuring that at least one 3′ base of the primer matched the target locus only. The primers were consistently targeted at exon sequences and were then used to amplify target genes (or fragments of them) from 24 genetically diverse accessions selected on the basis of previously generated marker data (Berlin *et al*., 2014). The resulting amplicons were pooled per individual and custom‐barcoded during sequencing library preparation using the NEBNext kit (New England Biolabs, Ipswich, MA, USA). Libraries were sequenced on an Illumina GAIIx sequencer at the SNP&SEQ Technology Platform, Science for Life Laboratory, Uppsala University. As alignments to the *Populus* genome were possible only in conserved exon regions, a combined *de novo*‐reference approach was used to produce a reference sequence per gene. First, quality‐filtered (ConDeTri ver. 2.0, hq = 30, minL = 70, Smeds & Küstner, 2011) reads were aligned to the corresponding poplar sequence using Mosaik v1.1 (Lee *et al*., 2014). Next, contigs were constructed *de novo* using Velvet 1.04, again using only quality‐filtered reads. These were subsequently sorted by locus using blast (*e *< 10^−5^) and combined with regions of good fit to poplar to generate a single reference sequence per gene (PHRAP, www.phrap.org, kalign v2.04). Reads were then mapped to the references using Mosaik and SNPs called using samtools v0.1.18 (Li *et al*., 2009). Further details are given in Supplementary material and methods section A.

The resulting SNPs were then filtered for those present in at least two of the 24 screened accessions. SNPs that were at least 200 bp apart from one another, and had associated primer scores of 0.6 or greater (calculated using the Illumina Assay Design Tools, (ADT)), were retained as candidates for further genotyping. From SLU, 593 candidate SNPs were selected for genotyping together with 175 additional SNPs used in a previous linkage study of Berlin *et al*. (2010). Genotyping of the association mapping population was performed using the Illumina Golden Gate Assay at the previously mentioned SNP&SEQ Technology Platform.

At RRes, candidate gene homologues were first identified using the *P. trichocarpa* genome sequence and the best poplar gene hit then used in a BLASTN search against a preliminary genome assembly of *S. viminalis* genotype NWC663 (unpublished data). Positive willow contigs were then aligned to their orthologous poplar gene model and used to design primers (using Primer3) for PCR amplification of the candidate gene set in the same set of 24 diverse *S. viminalis* accessions as used at SLU. Where possible, primers were designed in untranslated regions of the gene to minimize the amplification of paralogous loci. A maximum amplicon size of 5 kb was used, with multiple primer sets designed for longer genes if required. Preformulated PCR Master Mix (Promega, Madison, WI, USA) was used for all PCRs with primers synthesized and desalted by Sigma. Following amplification, products were run on a 1% agarose gel and bands of the expected size were excised and gel‐purified using QIAquick gel extraction kits in 96‐cell format (Qiagen, Hilden, Germany). Eluted DNA from all successfully amplified genes was then pooled in equimolar amount per individual, barcoded and sequenced on an Illumina GAIIx sequencer at the University of Bristol Genomics Facility. Reads were demultiplexed, mapped back to the target reference sequences using the Bowtie short‐read aligner (Langmead *et al*., 2009) with default parameters and SNPs identified by manual inspection of the resulting 24 alignments in the Geneious software.

For genotyping, 768 SNPs were selected based on a requirement for an associated Illumina iCom score greater than 0.6 and a representative spread across the target gene space. Genotyping of the association mapping population individuals (Illumina Goldengate Assay) was then carried out at the Genome Centre, Barts and The London, Queen Mary's School of Medicine and Dentistry, London. In addition to the SNPs, genotype data from 35 microsatellite (SSR) markers previously developed (Trybush *et al*., 2012) were also used for the purpose of kinship matrix estimation.

### Genetic analysis

Association mapping was carried out using a three‐step approach (Fig. 1). First, genetic structure parameters such as proportions of population ancestry and individual kinship were estimated using genotypic marker data only. Second, accession (clonal) estimators based on all phenotypes of each accession, but adjusted for systematic and random environmental variation, were developed. Subsequently, the accession estimators were used as variables dependent on the genetic design terms in an association mapping linear mixed model.

#### Genetic structure and kinship analysis

For the association mapping analysis, effects due to genetic population structure and fine‐scale kinship between individuals had to be taken into account. Therefore, population structure was inferred from the 35 SSRs genotypic data using the software STRUCTURE (Pritchard *et al*., 2000a). The sample comprised four distinct populations roughly localized to eastern Europe, western Europe, Sweden and western Russia (see Berlin *et al*., 2014). Moreover, because an admixture model was used, all accessions were individually assigned ancestry proportions to each population arranged in a population ancestry covariate matrix **F**. From previous genotype investigations, it was evident that some of the sampled accessions were genotypically identical, probably due to natural clonal propagation and historical long‐distance trade (Berlin *et al*., 2014). Samples in such groups were consequently collapsed into single accessions leaving 323 unique accessions for further analysis. As the field trial establishment was performed before this was known, several unique accessions were, in fact, represented by a higher number of plants/measurements than the six original replicates. Also, a kinship matrix (**K**) for the sample population was estimated using the genotypic data for the 35 SSRs and all SNPs that were polymorphic (1377) jointly (Fig. S1). **K** was estimated in accordance with Loiselle *et al*. (1995) using the SPaGeDi software (Hardy & Vekemans, 2002). The raw matrix was linearly rescaled to only contain positive values ranging from zero to the theoretical maximum of 1.

#### Development of accession estimators

Prior to analysis, phenotypic traits were checked for substantial deviations from a normal distribution and for variance heteroscedasticity. Such deviations were observed for GC10, SumD11 and Nsh11 at Pustnäs and LS10 at Woburn. These traits were therefore square‐root‐transformed. Subsequently, environmentally adjusted accession estimators were obtained by fitting linear mixed models (LMM) to the phenotypic data:

(1) $$y=X_{b}b+X_{c}c+p+e$$

where **y** is the phenotypic observation vector of all plants, **b** and **c** are the vectors of fixed block and accession (clone) effects, respectively, **p** is the vector of random effects of two‐dimensional spatial position, and **e** is the random residual. The design matrices **X**_*b*_ and **X**_*c*_ link the respective block and accession effect vectors to their corresponding elements in the observation vector. All effects were considered to be independent except for the spatial position effects (**p**), which were set to follow a two‐dimensional first‐order autoregressive correlation structure (Cullis & Gleeson, 1991) across plant rows and columns for all the Pustnäs data. For the Woburn trial, only for BB10, a one‐dimensional first‐order autoregressive correlation structure, across columns, was required (see Berlin *et al*., 2014 for further detail). Model parameter estimation for eqn (1) was carried out using a residual maximum likelihood (REML) method as implemented in genstat (©VSN International Ltd, Hemel Hempstead, UK) and asreml (Gilmour *et al*., 2009).

When developing the best linear unbiased estimators (BLUE) for the accessions, **c** was considered to be fixed rather than random because the accessions originated from genetically and phenotypically divergent populations. Accession distributions may thus deviate considerably from the normal distribution, thereby violating an assumption on which the prediction of random effect relies (Searle *et al*., 1992). However, in addition to the estimators *per se*, the (broad sense), accession estimator heritability ($H_{c}^{2}$) was also estimated for each trait and for that purpose **c** was instead considered to be random. $H_{c}^{2}$ may serve as an indicator of accession estimator precision and was calculated from the estimated accession variance ($\sigma_{c}^{2}$) and error variance ($\sigma_{e}^{2}$) components as:

(2) $$H_{c}^{2}=\frac{\sigma_{c}^{2}}{\sigma_{c}^{2}+\frac{1}{n}\sigma_{e}^{2}}$$

where *n* is the harmonic mean of the number of measured plants per accession.

#### Association mapping analysis

Combining the accession estimator and the estimated trait mean ($\hat{\mu }$) into a new observation variable ($y_{\mathrm{es}}=1\hat{\mu }+\hat{c}$), a second series of LMM analyses was conducted. For this, 316 and 322 successfully genotyped and unique accessions, present at Pustnäs and Woburn, respectively, were included (315 in common). Also the SNPs were further checked with respect to their minor allele frequency (MAF) and to the LD between markers which was estimated using a maximum likelihood method (Hill, 1974) implemented in the function ‘LD’ in the *Genetics* library of R. Only SNPs with a MAF > 5% and only one representative SNP out of groups of SNPs in complete LD were retained for association analysis. All the retained 1233 SNPs were biallelic. Linear mixed models based on that of Yu *et al*. (2006) were applied in separate analyses for each site and each retained SNP marker as:

(3) $$y_{\mathrm{es}}=\mathrm{Fq}+\mathrm{Sg}+\mathrm{Zu}+e_{\mathrm{es}}$$

where **q** and **g** are the vectors of fixed population ancestry and SNP genotype effects, respectively, **u** is the vector of random additive genetic effects that stems from marker‐based individual kinship (*chip* additive genetic effects), and **e**_es_ is the vector of the random residual effects. The design matrix **S** constitutes the individual genotypes of the analysed SNP as separate and independent factors, implying genetic effects of the form $g={\left[g_{AA}\;g_{Aa}\;g_{aa}\right]}^{T}$ for the genotypes *AA*,* Aa* and *aa,* respectively. **F** and **Z** link the respective individual ancestry proportion and additive chip effect to its corresponding observation. All effects were considered to be statistically independent except for the chip additive genetic effects whose variance was assumed to be structured as $\text{Var}\left(u\right)=2\sigma_{A}^{2}K$ where $\sigma_{A}^{2}$ is the chip additive genetic variance and **K** is the kinship matrix. Due to unsuccessful individual genotyping, 3.8% of the genotype data in **S** was missing for the average SNP (see Table S1 for details). Such occurrences were dealt with by excluding records with missing genotypes from analysis. Association mapping analysis (Eqn (3)) was conducted through REML analysis using in parallel the two softwares tassel (Bradbury *et al*., 2007) and asreml. The percentage of variance explained by the SNP (*R* ^2^) was estimated through TASSEL. Eventual inflation of test statistics as a result of unaccounted population structure and/or kinship was assessed by examining the dependency between observed *P*‐values sorted by magnitude and the corresponding expected *P*‐values under the null hypothesis of no association (QQ‐plots, Fig. S2).

For association analysis of SNPs, the full model (Eqn (3)) was used, but to assess the overall percentage of inheritable trait variation, additional analyses were made excluding any individual SNP effects (**Sg**) from the model. Thus, for each trait, the narrow‐sense chip heritability adjusted for population structure ($h_{s}^{2}$) was calculated from the estimated chip additive genetic and residual variance components ($\sigma_{e,\mathrm{es}}^{2}$) as:

(4) $$h_{s}^{2}=\frac{\sigma_{A}^{2}}{\sigma_{A}^{2}+\sigma_{e,\mathrm{es}}^{2}}$$

To address the possibility that either of the **Fq** or **Zu** terms were superfluous, these were subjected to significance testing omitting the individual SNP term (see Supplementary material and methods section B). For the traits where the tests indicated a reduced model to be preferable (see Table S2), we used that model to redo the association mapping analysis. The results were, however, very similar to those of the full model, and for the sake of consistency, only the full model association results are further treated. Furthermore, to estimate accession correlations between traits (*r*_*s*_) thereby assessing the occurence of genotype‐by‐environment (G × E) interactions, multivariate model analyses (extensions of eqn (3)) were performed treating each trait‐year‐site combination as a separate variate (e.g. Burdon, 1977). In a similar fashion, it was also possible to formally test whether SNP–trait associations were *common* across sites and assessment years and/or they showed *interactions* with sites and years (Korte *et al*., 2012). See Supplementary material and methods section C for more details. Estimation errors for all genetic parameters were calculated using the delta method based on the Taylor series approximation (Casella & Berger, 2002).

#### Association significance testing

Significance testing of SNP–trait associations was performed using the Wald *F*‐test. The inclusion of false positives due to multiple testing was controlled by assessing the false discovery rate quotient (*FDR‐q*, Storey & Tibshirani, 2003) for each SNP–trait combination. *FDR‐q* values were calculated by subjecting all nominal Wald *F P*‐values, for each trait separately, to the function ‘qvalue’ in R in order to estimate the overall proportion of true null hypotheses (*π*_*0*_) and thus to obtain *q*‐values. The *smoother* option of ‘qvalue’ was used, and the lower cut‐off (*λ*) values were set within the range 0–0.8 with a step size of 0.1 to keep the variance of *π*_*0*_ estimates at reasonably low levels (Storey & Tibshirani, 2003). SNPs with *q*‐values in the range 0.05–0.2 were considered here to be *suggestively* associated, while SNPs with *q *<* *0.05 were considered *significantly* associated with the trait in question.

#### Adjusting for threshold selection bias

It is known that markers subjected to intensive selection based on hypothesis test values frequently show severely overestimated *R* ^2^ and effects (**ĝ**) when reported in isolation (Beavis, 1994; Xu, 2003). This bias is often called *threshold selection bias*,* Beavis effect* or *winner's curse*. To assess the severity of this bias, additional univariate analyses were made on sets of simulated accession estimator data (**y**_*si*_). Simulated data were designed to mimic the presence of artificial SNP effects with a *prespecified* percentage of explained variance but with the objective of re‐estimating this parameter based on the simulated data regardless of the prior knowledge (Allison *et al*., 2002). Thus, *threshold bias‐adjusted* ratios of variance explained ($R_{\mathrm{adj}}^{2}$) and corresponding SNP effects (${\hat{g}}_{\mathrm{adj}}$) were estimated (see Supplementary material and methods D and E). Using the data from these simulations, the statistical power for detecting SNP–trait associations at FDR = 0.2 given $R_{\mathrm{adj}}^{2}$ values in the range of 0–10% was also estimated for each trait.

### Postanalysis verification of SNPs

To verify the rare genotypes for the two most prominently associated SNPs (ELF3b‐5128 and FLD‐1186), resequencing of fragments covering these loci was performed for ten and six association mapping accessions, respectively, that represented all three possible genotype classes. The DNA sequences containing ELF3b‐5128 and FLD‐1186 were aligned to the recently available *Salix purpurea* genome using blast (*Salix purpurea* v1.0, DOE‐JGI, http://phytozome.jgi.doe.gov/pz/portal.html#! info? alias = Org_Spurpurea), and the best hits were located in SapurV1A.1320s0030 and SapurV1A.0213s0120, respectively. Based on these *S. purpurea* sequences, new PCR primers were designed (Primer3Plus software, Untergasser *et al*., 2007) to cover the SNP sequences, and PCR using these primers was carried out. Products were separated on 1% agarose gels to confirm fragment size, followed by Sanger sequencing (Macrogen Inc., Seoul, Korea). SNP genotypes were then confirmed using the SeqMan program in Lasergene (DNASTAR Inc., Madison, WI, USA).

## Results

All traits for spring and autumn phenology were highly variable at both Pustnäs and Woburn during the different measurement years (Table 1). Less phenotypic variation was observed for bud burst during 2010 when the buds were regrowing from recently cutback stools. Considerable clonal/accession variation was also observed for all traits (Figs S3–S6). In general, the biomass traits, assessed in 2011, had higher mean values in Woburn compared to Pustnäs (Table 1). The large difference in summed diameter between sites was mainly a reflection of the considerable differences in the number of shoots rather than the differences in mean and maximum shoot diameters, which were modest.

### Genetic parameters

Broad‐sense accession estimator heritabilities ($H_{c}^{2}$) and narrow‐sense chip heritabilities ($h_{s}^{2}$) are presented in Table 2. The autumn phenology traits (GC10, LS10 and LS11) exhibited the highest $H_{c}^{2}$ estimates (0.88–0.96), indicating excellent estimator precision. The corresponding $H_{c}^{2}$ estimates for bud burst in spring and biomass traits were also generally high (0.55–0.93) except for bud burst at Woburn 2010 (0.24). Several $h_{s}^{2}$ estimates were also substantial (10 of 17 higher than 0.2), indicating that the marker‐estimated additive kinship term explained appreciable proportions of the population structure‐adjusted accession variation. Structure‐adjusted accession correlations between Pustnäs and Woburn (Table 2) were only moderate (0.32–0.61) and were markedly lower than unity with respect to the estimation errors. This indicates substantial G × E interactions that cannot be attributed to across‐trial differences in variance (e.g. Figs S3, S6). Accession trait–trait correlations within trials varied considerably depending on the traits studied (Table 3). Correlations across years for spring and autumn phenology traits varied from weak to moderately positive (0.10–0.55). In contrast, highly positive correlations were observed between the growth trait pairs Nsh11‐SumD11 and MeanD11‐MaxD11 (0.83–0.89). Regarding the population kinship, pairwise kinships estimated from all polymorphic markers were generally low (*θ *< 0.05, Fig. S1) and were noticeably higher within the subpopulation clusters indicated by the previous study (Berlin *et al*., 2014). In 193 cases (0.4% of all pairs), there were indications of closer kinship (*θ *> 0.2).

**Table 2:** Broad‐sense accession estimator heritabilities ($H_{c}^{2}$) and structure‐adjusted narrow‐sense chip heritabilities ($h_{s}^{2}$) estimated for each trait measured at Pustnäs and Woburn along with the structure‐adjusted accession correlations (*r*_*s*_) between these two field trials

Trait	Pustnäs		Woburn		*r*_*s*_
	$H_{c}^{2}$	$h_{s}^{2}$	$H_{c}^{2}$	$h_{s}^{2}$	
Spring phenology traits					
BB10	0.65 (0.03)	0.08 (0.11)	0.24 (0.06)	0.01 (0.09)	0.32 (0.05)
BB11	0.84 (0.01)	0.37 (0.11)	0.93 (0.01)	0.23 (0.11)	0.49 (0.05)
BB13	0.80 (0.02)	0.33 (0.10)	–	–	–
Autumn phenology traits					
GC10	0.91 (0.01)	0.40 (0.11)	–	–	–
LS10	0.94 (0.01)	0.41 (0.11)	0.96 (0.00)	0.26 (0.12)	0.39 (0.06)
LS11	0.88 (0.01)	0.04 (0.11)	–	–	–
Biomass growth traits					
Nsh11	0.55 (0.04)	0.10 (0.10)	0.71 (0.03)	0.14 (0.08)	0.58 (0.04)
MeanD11	0.67 (0.03)	0.42 (0.10)	0.79 (0.02)	0.41 (0.08)	0.61 (0.04)
MaxD11	0.72 (0.02)	0.38 (0.09)	0.79 (0.02)	0.37 (0.09)	0.54 (0.04)
SumD11	0.55 (0.04)	0.04 (0.09)	0.70 (0.03)	0.15 (0.09)	0.53 (0.04)

Estimation errors are given in parentheses.

**Table 3:** Accession correlations (*r*_*s*_) adjusted for population structure between traits measured in Pustnäs (above diagonal) and Woburn (below diagonal)

Trait	BB10	BB11	BB13	GC10	LS10	LS11	Nsh11	MeanD11	MaxD11	SumD11
BB10	x	**0.42**	**0.24**	**−0.19**	**−**0.08	**−**0.01	**0.29**	0.10	**0.16**	**0.36**
BB11	**0.19**	x	**0.55**	**−0.14**	**−0.14**	0.03	0.05	**0.32**	**0.33**	**0.22**
BB13			x	0.00	**−**0.03	0.04	0.01	0.08	0.12	0.02
GC10				x	**0.46**	0.10	**−**0.07	0.06	0.12	**−**0.09
LS10	**−**0.10	**−0.18**			x	**0.50**	**−**0.01	**0.19**	**0.22**	0.08
LS11						x	0.12	**0.26**	**0.27**	**0.23**
Nsh11	0.11	0.04			**−**0.03		x	**−0.16**	0.08	**0.83**
MeanD11	0.09	**−**0.08			0.08		0.00	x	**0.86**	**0.32**
MaxD11	0.10	**−**0.07			0.10		**0.19**	**0.87**	x	**0.49**
SumD11	**0.15**	0.01			**−**0.01		**0.89**	**0.43**	**0.55**	x

Estimate magnitudes twice the size of their standard errors are given in bold.

### Association patterns and stability

In univariate analyses, 11 significant (FDR level at 0.05) and 23 suggestive (FDR level at 0.2) SNP–trait associations were detected encompassing in total 29 different SNPs (Table 4). The leaf senescence traits exhibited the greatest number of associations (19), whereas fewer associations were observed for spring bud burst traits (10) and biomass traits (5) despite the fact that the number of assessments for the leaf senescence traits across years and field trials (3) was lower than the corresponding number of assessments for bud burst (5) and biomass (8). No associations were observed for days to growth cessation (GC10) or for summed shoot diameter (SumD11). With only LS10 at Woburn as a possible exception, no obvious signs of test statistic inflation were detected as the low end of observed and sorted ‐log(*p*)‐values fell very close to the line of identity (Fig. S2). The percentage variance explained (*R* ^2^) ranged from 1.4% to 7.8% for all associations of at least suggestive nature, but ranged only from 0.3% to 4.4% when adjusted for threshold selection bias ($R_{\mathrm{adj}}^{2}$).

**Table 4:** SNP–trait associations of suggestive significance (*FDR‐q *<* *0.2) observed from univariate analysis of the Pustnäs or Woburn trials are listed with their nominal *P*‐values, false discovery rate quotients (*q*) and the percentage of accession predictor variance explained by the SNP in the respective trial unadjusted (*R* ^2^) and adjusted ($R_{\mathrm{adj}}^{2}$) for threshold selection bias

SNP	Pustnäs				Woburn			
	*P*	*q*	*R* ^*2*^ (%)	$R_{\mathrm{adj}}^{2}$ (%)	*P*	*q*	*R* ^*2*^ (%)	$R_{\mathrm{adj}}^{2}$ (%)
Bud burst 2010								
ELF3b‐5128	2.0 × 10^−4^	0.1129	4.9	1.0	4.6 × 10^−6^	**0.0055**	7.8	2.4
APR3‐2085	1.6 × 10^−4^	0.1129	5.2	1.5	0.4483	0.9922	0.5	–
IX‐12‐sa‐pIII	3.2 × 10^−4^	0.1201	4.8	0.8	0.0070	0.8615	3.2	–
I‐6om‐sa	0.6313	0.8631	0.3	–	2.6 × 10^−4^	0.1631	5.4	0.6
X‐13‐sa	6.7 × 10^−4^	0.1886	4.6	0.6	0.1226	0.9707	1.5	–
Bud burst 2011								
PU08629‐458	1.2 × 10^−4^	0.0692	5.7	2.1	0.0457	0.7546	1.8	–
DT827847‐504	1.9 × 10^−4^	0.0692	5.4	1.7	0.3497	0.9317	0.6	–
ELF4a‐288	1.7 × 10^−4^	0.0692	5.3	1.6	0.0469	0.7546	1.7	–
Bud burst 2013								
ELF3b‐5128	4.7 × 10^−5^	0.0535	6.4	1.5	–	–	–	–
Leaf senescence 2010								
FLD‐1186	0.5056	0.9376	0.3	–	9.1 × 10^−7^	**0.0004**	4.6	4.4
SBP1‐3964	0.5862	0.9471	0.2	–	3.7 × 10^−7^	**0.0004**	4.4	4.1
ZIP1‐4494	0.0700	0.8708	1.1	–	8.9 × 10^−7^	**0.0004**	4.1	3.8
APS1‐203	0.3275	0.9111	0.5	–	1.6 × 10^−5^	**0.0046**	3.2	2.6
PU07550‐3852	0.5323	0.9458	0.3	–	1.9 × 10^−5^	**0.0046**	2.8	2.1
PtPHYB2‐3897	0.0118	0.7315	2.0	–	3.6 × 10^−5^	**0.0072**	3.1	2.5
ELF3b‐5128	0.6601	0.9612	0.2	–	4.4 × 10^−5^	**0.0076**	2.3	1.0
PtFT1‐340	0.8281	0.9676	0.1	–	1.3 × 10^−4^	**0.0193**	2.8	2.1
APR1‐2372	0.5572	0.9471	0.2	–	3.8 × 10^−4^	0.0514	2.1	1.4
MPS3‐253	0.5127	0.9376	0.3	–	8.7 × 10^−4^	0.0807	2.2	1.5
UNF2‐1375	0.8509	0.9684	0.1	–	8.5 × 10^−4^	0.0807	2.1	1.4
UNF2‐1793	0.6706	0.9612	0.2	–	6.8 × 10^−4^	0.0807	2.0	1.3
DT1122704‐744	0.0397	0.8708	1.4	–	7.8 × 10^−4^	0.0807	1.9	1.2
PU08382‐47	0.1004	0.8708	1.0	–	0.0012	0.1041	1.5	0.5
PMI1‐1126	0.3170	0.9111	0.5	–	0.0017	0.1387	1.7	0.7
NCD3‐3144	0.2290	0.8736	0.6	–	0.0020	0.1532	1.5	0.5
VI‐7om‐sa	0.6222	0.9612	0.2	–	0.0027	0.1940	1.4	0.3
Leaf senescence 2011								
R‐32‐sa‐pI	2.1 × 10^−4^	0.1302	4.1	1.9	–	–	–	–
VII‐17‐sa‐pIII	2.0 × 10^−4^	0.1302	2.8	0.3	–	–	–	–
No. shoots 2011								
QTL10‐4‐179	6.4 × 10^−5^	0.0771	6.1	2.1	0.1695	0.9243	1.1	–
X‐f17‐2	2.8 × 10^−4^	0.1326	5.2	0.6	0.0358	0.8926	2.1	–
PU12538‐759	3.3 × 10^−4^	0.1326	5.0	0.6	0.1287	0.9036	1.3	–
Mean shoot diameter 2011								
FLD‐1186	4.2 × 10^−5^	**0.0465**	6.4	0.6	0.0916	0.9364	1.4	–
Maximum shoot diameter 2011								
FLD‐1186	2.2 × 10^−5^	**0.0257**	6.6	0.7	0.0614	0.8174	1.6	–

*q*‐values below 0.05 are highlighted in bold.

Among the 29 SNPs suggestively associated with traits (*q *<* *0.2) in univariate analyses, only ELF3b‐5128 was found to be suggestively associated with the same trait (bud burst) at both Pustnäs and Woburn and at more than one assessment year (2010 and 2013, Table 4). However, when regarding multivariate analyses, ten of 19 trait‐associated SNPs overlapped with those of univariate associations (Table 5). Seven of these overlapping SNPs moreover showed associations common across sites and/or years (*P *<* *0.001), indicating a degree of association consistency (APS1‐203, DT827847‐504, ELF3b‐5128, FLD‐1186, PtPHYB2‐3897, SBP1‐3964 and ZIP1‐4494). Nonetheless, even accounting for the multivariate analysis, the overall association pattern was not very consistent, especially as nine of the 19 associated SNPs exhibited associations interacting with years and/or sites (*P *<* *0.001), thus supporting the existence of G×E interactions at the level of individual SNPs.

**Table 5:** Suggestive SNP–trait associations (full model *FDR‐q *<* *0.2) from multivariate analyses across sites and years (variates) are listed with their nominal *P*‐values for full model, common model and interaction model tests

SNP	Full *q*	Full *P*	Common *P*	Interaction *P*
Bud burst, 5 variates				
ELF3b‐5128^a^	0.0002	**1.5 × 10** ^−**7**^	**2.8 × 10** ^−**5**^	**9.2 × 10** ^−**5**^
DT827847‐504^a^	0.0718	**1.8 × 10** ^−**4**^	**2.7 × 10** ^−**4**^	0.0223
PU08629‐1539	0.0718	**2.6 × 10** ^−**4**^	**1.1 × 10** ^−**4**^	0.0479
QTL10‐8‐190	0.0718	**2.8 × 10** ^−**4**^	0.0120	0.0019
MYB1‐496	0.0753	**3.7 × 10** ^−**4**^	0.6988	**1.5 × 10** ^−**4**^
PU12382‐2407	0.0860	**5.1 × 10** ^−**4**^	0.1189	**5.6 × 10** ^−**4**^
APR3‐2085^a^	0.1741	0.0012	0.0116	0.0080
Leaf senescence, 3 variates				
ZIP1‐4494^a^	0.0045	**7.0 × 10** ^−**6**^	**6.5 × 10** ^−**5**^	0.0035
SBP1‐3964^a^	0.0045	**1.1 × 10** ^−**5**^	**4.8 × 10** ^−**5**^	0.0081
FLD‐1186^a^	0.0045	**1.2 × 10** ^−**5**^	0.0016	**4.7 × 10** ^−**4**^
PtPHYB2‐3897^a^	0.0274	**9.4 × 10** ^−**5**^	**1.6 × 10** ^−**5**^	0.1907
APS1‐203^a^	0.0904	**3.9 × 10** ^−**4**^	**1.4 × 10** ^−**4**^	0.1232
PU07550‐3852^a^	0.0932	**4.8 × 10** ^−**4**^	0.0011	0.0272
PtFT1^a^	0.1718	0.0010	0.0128	0.0075
Mean shoot diameter, 2 variates				
FLD‐1186^a^	0.0942	**8.1 × 10** ^−**5**^	0.0070	**5.9 × 10** ^−**4**^
Maximum shoot diameter, 2 variates				
VII‐3b	0.0999	**1.4 × 10** ^−**4**^	0.0561	**1.9 × 10** ^−**4**^
APR1‐748	0.0999	**1.7 × 10** ^−**4**^	0.7121	**2.3 × 10** ^−**5**^
FLD‐1186^a^	0.1537	**3.9 × 10** ^−**4**^	**9.3 × 10** ^−**4**^	0.0255
IFR1‐2076	0.1547	**5.2 × 10** ^−**4**^	0.1011	**4.3 × 10** ^−**4**^
HRD2‐855	0.1557	**6.5 × 10** ^−**4**^	0.0916	**6.3 × 10** ^−**4**^
Summed shoot diameter, 2 variates				
PU08629‐5000	0.1213	**1.0 × 10** ^−**4**^	0.2209	3.2 × 10^−5^

*P‐*values below 0.001 are highlighted in bold.

a Also detected (*q *<* *0.2) in univariate association analyses.

Assessments of the statistical power for association detection at *q *<* *0.2 (Fig. S7) showed that a hypothetical true SNP–trait association exhibiting an $R_{\mathrm{adj}}^{2}$ of 2% would unlikely be detected in any of the traits studied (power<0.22) except only for LS10 in Woburn (power ≈ 0.72). The fact that 17 associations of such, or even lower, $R_{\mathrm{adj}}^{2}$ magnitudes nonetheless were detected for traits other than LS10 suggests that there may be several more small magnitude trait associations among our candidate SNPs that were not detected in this analysis. To some extent, the limited statistical power at the $R_{\mathrm{adj}}^{2}$ level of 2% is also consistent with the poor association consistency observed across trials in univariate analyses.

### Significant and repeatedly observed associations

**Figure 2:**
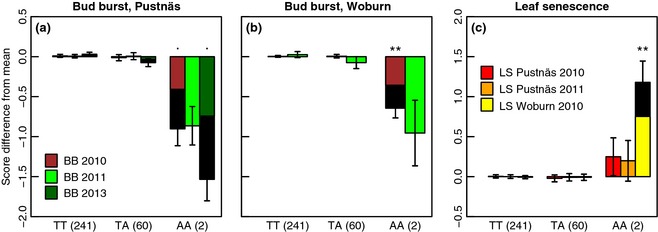
Genotype effects for SNP ELF3b‐5128 on bud burst at Pustnäs (a), Woburn (b) and on leaf senescence (c) at both trials. Effects connected with considerable likelihood of true association are marked with: . *q *<* *0.2 and **q <* *0.01. The coloured part of the staples show threshold selection bias‐adjusted effects, while the original biased effect is shown as black staples in the background. Effects for transformed traits are given in the back‐transformed scale, and the values in parentheses after each genotype group describe the number of accessions of that group.

Among the SNP–trait associations, the SNP ELF3b‐5128 (*EARLY FLOWERING 3*) was suggestively or significantly associated with bud burst for two measurements at Pustnäs and one at Woburn (Table 4) and was also significantly associated in the multivariate analysis (Table 5). ELF3b‐5128 explained 1.0–2.4% of accession variation when adjusted for threshold selection bias. Correspondingly, accessions with the rare *AA* genotype of this SNP were observed to have consistently and substantially lower bud burst (BB) scores (by 0.4–1.0) compared to the overall mean in both trials (Fig. 2a,b), indicating a slower or delayed bud burst process of the *AA* genotypes in the spring. The association pattern observed agreed with ELF3b‐5128, showing both common and interaction associations (*P *<* *0.001) with bud burst in the multivariate analysis because the direction of the SNP effect was consistent across years and sites, while the effect magnitudes appeared to vary. The same rare ELF3b‐5128 genotype was also associated with higher LS10 scores (by 0.2–0.8) compared to the overall mean (Fig. 2c), but this association was significant only for the univariate analysis at Woburn.

**Figure 3:**
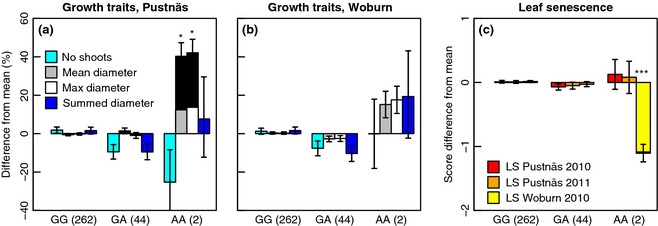
Genotype effects for SNP FLD‐1186 on growth traits at Pustnäs (a), Woburn (b) and on leaf senescence (c) at both trials. Effects connected with considerable likelihood of true association are marked with: *q <* *0.05 and ***q <* *0.001. The coloured part of the staples show threshold selection bias‐adjusted effects, while the original biased effect is shown as black staples in the background. Effects for transformed traits are given in the back‐transformed scale, and the values in parentheses after each genotype group describe the number of accessions of that group.

The SNP FLD‐1186 (*FLOWERING LOCUS D*) was significantly associated with mean and maximum shoot diameter at Pustnäs and with leaf senescence at Woburn (Table 4) and was also observed to be associated with these traits in the multivariate analyses (Table 5). Even when adjusted for threshold selection bias, the rare *AA* genotype was associated with a 12% higher MeanD11 and a 14% higher MaxD11 at Pustnäs compared to the population average (Fig. 3a) corresponding quite well to the nonsignificant FLD‐1186 *AA* effect estimates of MeanD11 and MaxD11 at Woburn (Fig. 3b). The FLD‐1186 *AA* genotypes also showed substantially lower LS10 values at Woburn than the other genotypes, while weak but opposite trends were visible at Pustnäs (Fig. 3c). This association pattern furthermore agreed with FLD‐1186, exhibiting a multivariate interaction‐type association with leaf senescence.

For the significant ELF3b‐5128, FLD‐1186 and other influential associations (e.g. PtPHYB2‐3897, SBP1‐3964 and ZIP1‐4494), genotypic effects appeared to be recessive in nature with respect to the rare allele and the genotype effects of the heterozygote and common allele homozygote appeared not to differ (Figs 2 and 3 and S8). This has important implications when comparing association $R_{\mathrm{adj}}^{2}$ with the different heritability estimates (Table 2). For instance, the $R_{\mathrm{adj}}^{2}$ estimate for the ELF3b‐5128 association with BB10 in Woburn (2.4%) should primarily be compared with the BB10 $H_{c}^{2}$ rather than $h_{s}^{2}$, as dissection of the ELF3b‐5128 genotypic effects showed that ELF3b‐5128 makes a contribution to the additive genetic variance of less than 0.2%. Consequently, the $R_{\mathrm{adj}}^{2}$ estimates of rare allele recessive effect SNPs such as ELF3b‐5128 and FLD‐1186 are predominantly nonadditive also in terms of their genetic variance contribution.

### Linkage disequilibrium

By studying the effects of SNPs in strong LD (*r* ^2^ **>* *0.5) with the observed suggestive or significant associations, it was possible to evaluate whether all our associations could be considered as independent or whether any of the associations could be (partially) explained by alternative SNPs. For eight associations within the genes *DT827847*,* PU08629*,* DT1122704*,* APR1*,* NCD3*,* UNF2* and *ZIP*, eleven alternative SNPs were found to exhibit *P*‐values below 0.05 for the same type of trait, thus indicating association tendencies. All these alternative SNPs were in strong LD (*r* ^2^ in the range 0.53–0.97) with the original suggestive/significant associations and were also located within the same genes. In particular, the two *UNF2* SNPs both suggestively associated with LS10 in Woburn (Table 4) were found to be in strong LD with each other (*r* ^2^ **=* *0.90), thus likely reflecting one single association rather than two. LD estimates were consistently low (*r* ^2^ **≤* *0.16) between all other SNPs suggestively/significantly associated with traits, suggesting that they are much more likely to be independent than the UNF2 pair.

Nonetheless, when evaluating the potential association mapping resolution of the significant rare allele recessive effects exhibited by ELF3b‐5128 and FLD‐1186, estimates of whole population LD may not constitute an adequate measure as the extent of LD may be considerably greater for more closely related sets of individuals. For instance, the two accessions homozygous for the rare *A*‐allele of FLD‐1186 both belonged to the western Russian cluster and showed considerable kinship estimates (*θ *= 0.607), suggesting that the association might only be relevant in a rather limited genetic background. Moreover, this association may have been produced by one or several alternative loci situated at a greater distance than the population LD estimates for FLD‐1186 with other SNPs would indicate (*r* ^2^ **<* *0.13). On the other hand, the two accessions of the rare ELF3b‐5128 *AA* genotype belonged to different subpopulation clusters (eastern Europe and western Russia) and showed a very low kinship (*θ *= 0.028). The extent of LD for this particular association should not be greater than that suggested by the regular LD estimates (*r* ^2^ **<* *0.08) between ELF3b‐5128 and any other candidate SNP.

## Discussion

Previous QTL studies in *Salix* on adaptive growth and phenology traits have made substantial contributions to our knowledge about their genetic architecture. However, the usefulness of any markers detected by these studies for mas (Tsarouhas *et al*., 2002, 2003; Ghelardini *et al*., 2014) might be limited by the extensive LD in the QTL‐mapping populations. The *Salix* association mapping population investigated in this study comprised accessions originating from a great variety of locations throughout northern Europe, making the sample fairly representative for the germplasm available in the region. The results of Berlin *et al*. (2014) indicated that some of the studied traits, in particular leaf senescence, were strongly differentiated among populations (high *Q*_ST_ estimates), but the chip heritabilities for most traits were substantial even when this population structure was accounted for. Consequently, we took the approach of including both population structure parameters and fine‐scale individual kinship in our association mapping model to minimize the probability of detecting false‐positive associations.

To facilitate the study of G × E effects and conditional neutrality of SNPs, two quite contrasting sites were used in this study. The Swedish trial site (Pustnäs) is situated in an area in which many commercial willow bioenergy plantations are grown, and the growth data approximately reflect the production conditions in central Sweden. Although in an area of the south‐east UK which is predominantly arable for agriculture, the trial at Woburn presents an example of more challenging conditions due to the limited water‐ and nutrient‐holding capacity of the sandy, well‐drained soils at that site. However, the environmental conditions at the two trials were also different in photoperiod, temperature and precipitation, as well as soil texture (Berlin *et al*., 2014), and it was noted that the plants at Woburn grew faster on average than those at Pustnäs (Table 1), probably due to differences in latitude and temperatures.

### Association patterns and genetic parameters

Each SNP–trait association detected in this study explained only limited portions of the variation across accessions (<8%) and even less when selection threshold bias was taken into account (<5%). Similar observations have been made in several other association mapping studies in tree species (Eckert *et al*., 2009; Olson *et al*., 2013; Prunier *et al*., 2013; McKown *et al*., 2014). One should, however, be aware that the candidate gene approach pursued in the mentioned studies (including this one) is limited in the sense that a substantial portion of the genome diversity remains untested for the existence of major associations. Nonetheless, considering the generally high accession estimator and chip heritability estimates, the observations of this and most other studies suggest phenology and growth traits to be controlled by many loci with small individual effects.

Associations detected in the univariate analysis of one environment were only occasionally indicated to be stable across environments and years by multivariate analyses. Only one SNP (ELF3b‐5128) was observed to be suggestively/significantly associated across both trials, years and analysis methods. There are several reasonable explanations for the limited association consistency. First, it should be acknowledged that the criteria for multiple testing correction (*FDR‐q *<* *0.2) resulted in rather strict *P*‐value thresholds (consistently below 0.003, Table 4) for declaring an association suggestive or significant. This in combination with the likelihood of small true *R* ^2^ suggested by this study would result in few repeated observations in univariate analyses even assuming that the associations in truth would perfectly consistent across sites/years and accounting for the trait correlations. In addition, with respect to leaf senescence that showed the most conspicuous association inconsistencies, LS10 in Woburn exhibited QQ‐plots that indicated a possible minor association test statistic inflation. The previous genetic diversity study of this material showed an extremely strong population differentiation for leaf senescence, LS10 in Woburn being particularly extreme (*Q*_ST_ = 0.91, Berlin *et al*., 2014). Genetic studies in *Arabidopsis thaliana* have shown that an excess of false‐positive associations may be observed, despite careful modelling precautions given a situation where extensive population structure is combined with strong local trait adaptation (Aranzana *et al*., 2005; Zhao *et al*., 2007). This coincidence therefore suggests that some of the associations observed for LS10 in Woburn may be false positives despite their low *q*‐values, thus partly accounting for the association inconsistency. A third reasonable explanation for the weak association consistency is the substantial G×E interaction between Pustnäs and Woburn indicated by their relatively moderate accession correlations plus the detection of a number of SNP–trait associations of the interaction kind. For instance, the interaction‐type association between FLD‐1186 and leaf senescence would readily explain the presence of a strong FLD‐1186 association with LS10 in Woburn, while the corresponding association with LD10 and LS11 in Pustnäs was entirely absent indicating conditional neutrality (Fig. 3c).

Consequently, some of the observed association inconsistencies, low accession correlations across sites and the existence of significant interaction‐type associations suggest that certain SNP–trait associations detected in one environment may still not be usable as MAS markers for *Salix* in a very different one. This further highlights the need to test associations in multiple and differentiated environments using both univariate as well as multivariate analysis approaches (Korte *et al*., 2012). With additional data of this nature, MAS may even be performed using different sets of markers in multiple breeding populations each directed at different environments (Namkoong *et al*., 1988).

### Significant associations

Among the associations observed in this study, the SNPs ELF3b‐5128 and FLD‐1186 are notable as they showed suggestive/significant associations to several sites/traits and were further supported by exhibiting common‐type significant trait associations at least once in multivariate analyses. The existence of both SNPs and the veracity of their corresponding rare allele homozygous genotypes were verified by resequencing the corresponding fragments of these genes using the genome sequence of *S. purpurea* as a reference for primer design instead of *P. trichocarpa*. ELF3b‐5128 was found to be a nonsynonymous SNP located in the fourth exon of the *EARLY FLOWERING 3* gene on chromosome 3 according to blast searches in both *S. purpurea* and *P. trichocarpa* genome assemblies (see also Table S1). *ELF3* is involved in the circadian clock that is crucial for proper plant responses to changes in the photoperiod (Lagercrantz, 2009; Klintenäs, 2012 and references therein). In line with the results of this study, Ghelardini *et al*. (2014) observed a QTL for growth cessation that encompassed the location of *ELF3* in a cross between a *S. viminalis* and a *S. viminalis × schweriini* parent. Olson *et al*. (2013) also observed SNPs in *ELF3* to be significantly associated both with bud burst and bud set at several field trials of *P. balsamifera*. Furthermore, downregulation of circadian clock genes in *P. tremula × tremuloides* affects both timing of growth cessation and bud burst (Ibáñez *et al*., 2010). FLD‐1186 appeared to be a synonymous SNP located in the *FLOWERING LOCUS D* gene in chromosome 10 (*S. purpurea*) and also deserves further study. However, because the effect of this association stemmed from two accessions that were indicated with close kinship, causal factors situated far from the position of the *FLD* gene may have produced this association, thereby limiting the use of FLD‐1186 as a mas marker.

Even though percentages of accession variation explained by ELF3b‐5128 and FLD‐1186 were modest, some of the genotypic effects nonetheless appeared sufficiently great to be visible to the human eye even when adjusted for threshold selection bias. The explanation for this apparent contradiction is that the low minor allele frequencies (0.11 and 0.08 for the *A*‐allele for ELF3‐5128 and FLD‐1186, respectively) severely limit the extent to which even large genotype effects may explain the total accession variance (Lynch & Walsh, 1998). This is particularly true if the effect is recessive with respect to the rare allele. Consequently, MAS for the *A*‐allele of ELF3b‐5128 may help in selection of *S. viminalis* genotypes with delayed bud burst that could avoid damage caused by early spring frosts (Lennartsson & Ögren, 2004).

### Future research directions

Although the effects of the ELF3b‐5128 and FLD‐1186 *AA* genotypes were substantial and highly significant indicating usefulness in MAS, further validation is still warranted as they are based on very few accessions with the rare genotype. For this, multiple family QTL mapping (e.g. Ukrainetz *et al*., 2008) using crosses between parents heterozygous with respect to the SNPs in question may prove useful. In addition, transformation studies in model organisms such as poplar may also be of use.

One should also remember that the eventual usefulness of ELF3b‐5128 and FLD‐1186 in MAS does not imply their causality. Linkage disequilibrium in *S. viminalis* (Berlin *et al*., 2011) as well as in *P. trichocarpa* (Slavov *et al*., 2012) has been observed to extend to a range of 1 kbp or more. Moreover, several alternative SNPs in strong LD with associated SNPs of this study showed association tendencies *per se,* indicating that one and the same genotypic effect had been observed repeatedly. This suggests that other polymorphisms located in the proximity to the significantly associated SNPs candidate genes of this study may still hold the true causal effect in spite of our results.

Other SNPs that may warrant further investigation are those that exhibited inconsistent association pattern across trials or years in univariate analyses but still were observed to have multivariate common‐type trait associations (e.g. APS1‐203, DT827847‐504, PtPHYB2‐3897, SBP1‐3964, ZIP1‐4494 and SNPs in PU08629). This is particularly true for SNPs located in genes that have some relevant functional information. For example, PtPHYB2‐3897 was located in the orthologous coding sequence of the poplar *PHYTOCROME B2* gene (Table S1), which has been implicated in plant light perception (Smith, 2000) and shown to be associated with bud set in a population of *P. tremula* (Ingvarsson *et al*., 2008). In addition, a SNP in the poplar gene (*FT1*), known to be important for winter dormancy and flowering (Paul *et al*., 2014), was found to be associated with bud burst in *P. trichocarpa* (Evans *et al*., 2014), while in this study, the SNP PtFT1‐340, situated in the presumed *Salix* orthologue of *FT1,* was instead significantly associated with leaf senescence at Woburn.

In this study, some traits such as bud burst and leaf senescence were measured repeatedly. If such measurements were taken with greater regularity within each season, it would be possible to detect SNP associations with the temporal developments of each trait, thus taking into account their dynamic behaviour (e.g. Ma *et al*., 2002; Wu & Lin, 2006). Finally, the appreciably high chip heritabilities suggests that genomic selection based on many markers (Meuwissen *et al*., 2001; Nakaya & Isobe, 2012) could be possible even if particular SNP–trait associations are disregarded. Consequently, the potential of genomic selection also deserves further study to enhance and accelerate the future genetic improvement of *S. viminalis* for bioenergy and biofuel purposes.

## Supporting information


**Figure S1**. Heatmap of the kinship matrix.

**Figure S2.** QQ‐plots for all studied traits.

**Figure S3.** Histogram of the accession estimator distribution for bud burst across years and field trials.

**Figure S4.** Histogram of the accession estimator distribution for leaf senescence across years and field trials.

**Figure S5.** Histogram of the accession estimator distribution for growth cessation in 2010 at Pustnäs.

**Figure S6.** Histogram of the accession estimator distribution for growth traits assessed in 2011 in both field trials.

**Figure S7.** Probability of finding associations at the false discovery rate 0.2 for all traits in relation to association $R_{\mathrm{adj}}^{2}$.

**Figure S8.** Genotype effects of SNP ZIP1‐4494 (a), SBP1‐3964 (b) and PtPHYB2‐3897 (c) on leaf senescence at both trials.

**Supplementary Material and Methods**. (A) Salix reference sequence assembly, (B) Significance testing of structure model terms, (C) Multivariate analyses, (D) Adjusting for threshold selection bias by simulation and (E) Derivation of $R_{\mathrm{ps}}^{2}$.

**Table S1**. Characteristics for all SNPs included in the association mapping analysis.

**Table S2**. Significance tests of genetic structure model terms and suggested optimised model.
