# Supplementary material for: Association mapping in Salix viminalis L. (Salicaceae) – identification of candidate genes associated with growth and phenology
Source: Glob Change Biol Bioenergy. 2015 Jul 29;8(3):670–85. doi: 10.1111/gcbb.12280 (PMC4973673; doi:10.1111/gcbb.12280)

**Fig. S1** : Heatmap of the kinship matrix where accessions are sorted according to inferred subpopulation ancestry (W – Western Europe, S – Sweden, E – Eastern Europe, R – Western Russia, U – Mixed ancestry (<50% ancestry proportion in any particular subpopulation).


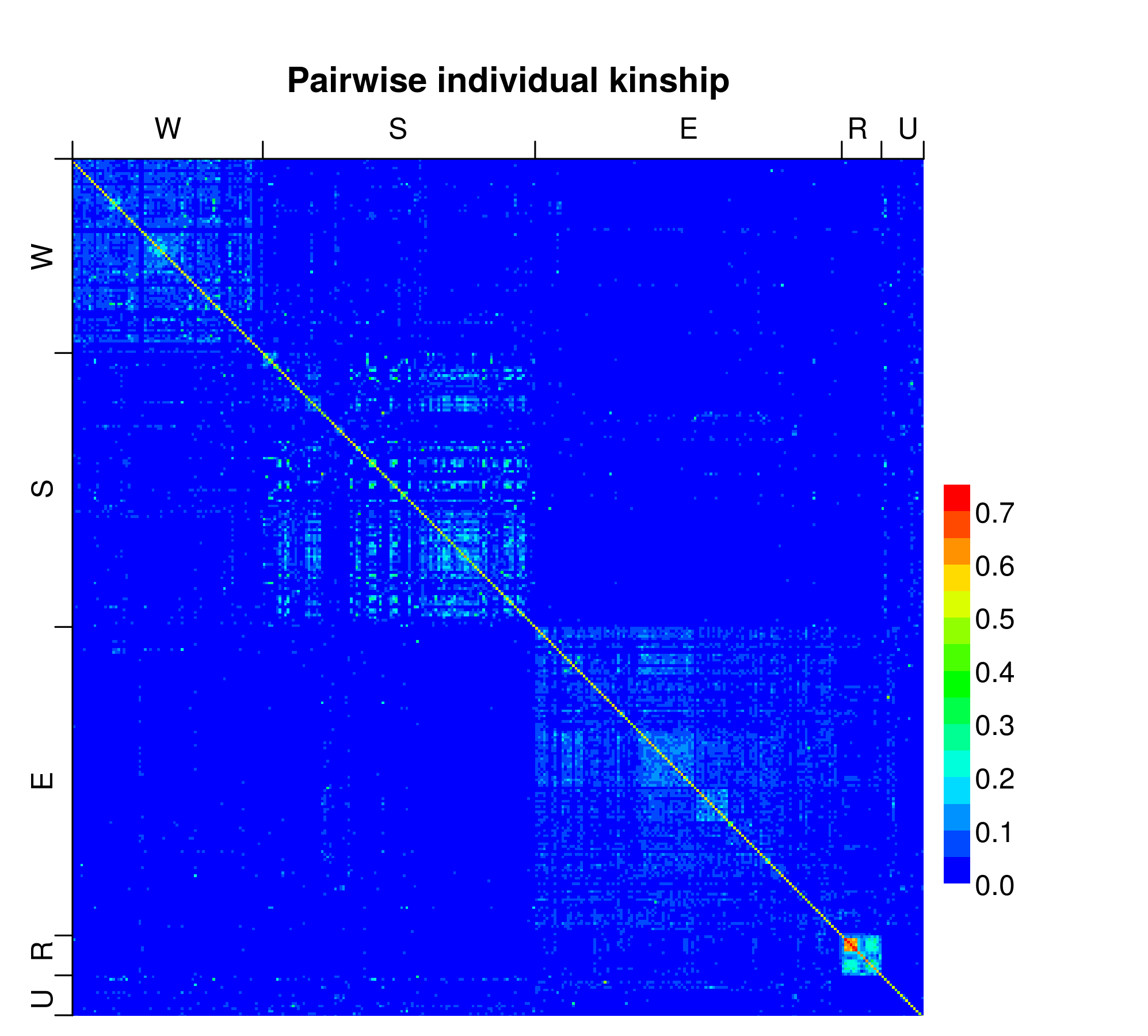

Supplement: Supplementary file 1 — Figure S1. Heatmap of the kinship matrix. [file GCBB-8-670-s001.docx]
