# Supplementary material for: Association mapping in Salix viminalis L. (Salicaceae) – identification of candidate genes associated with growth and phenology
Source: Glob Change Biol Bioenergy. 2015 Jul 29;8(3):670–85. doi: 10.1111/gcbb.12280 (PMC4973673; doi:10.1111/gcbb.12280)

**Fig. S2** : QQ-plots for all studied traits showing the dependency between observed sorted SNP association *p*-values and expected *p*-values under the null-hypothesis of no associations. Values are given in negative logarithmic scale.


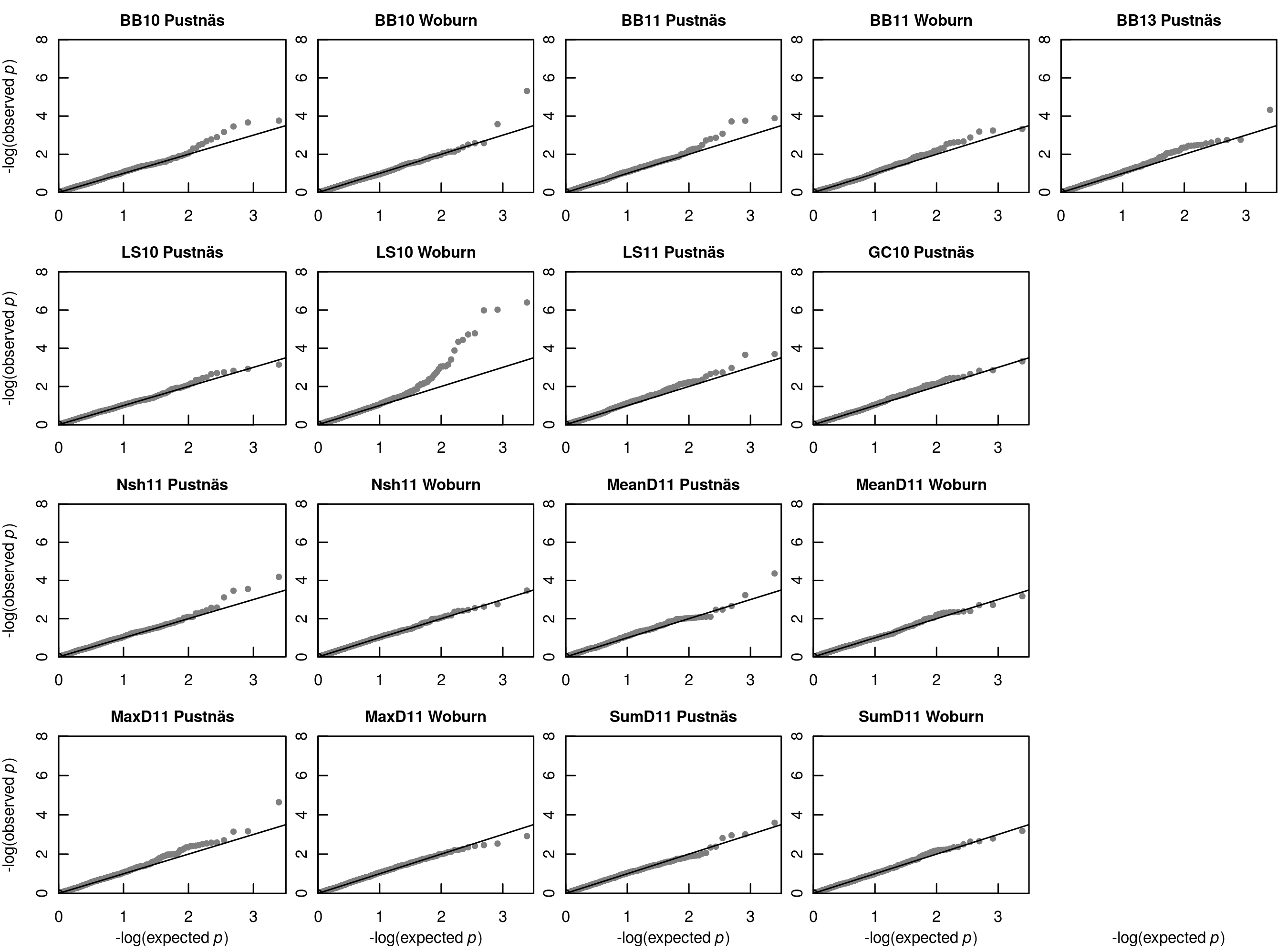

Supplement: Supplementary file 2 — Figure S2. QQ‐plots for all studied traits. [file GCBB-8-670-s002.docx]
