# Supplementary material for: Association mapping in Salix viminalis L. (Salicaceae) – identification of candidate genes associated with growth and phenology
Source: Glob Change Biol Bioenergy. 2015 Jul 29;8(3):670–85. doi: 10.1111/gcbb.12280 (PMC4973673; doi:10.1111/gcbb.12280)

**Fig. S3** : Histogram of the accession estimator distribution for bud burst across years and field trials showing the assessment date in the upper right corner.


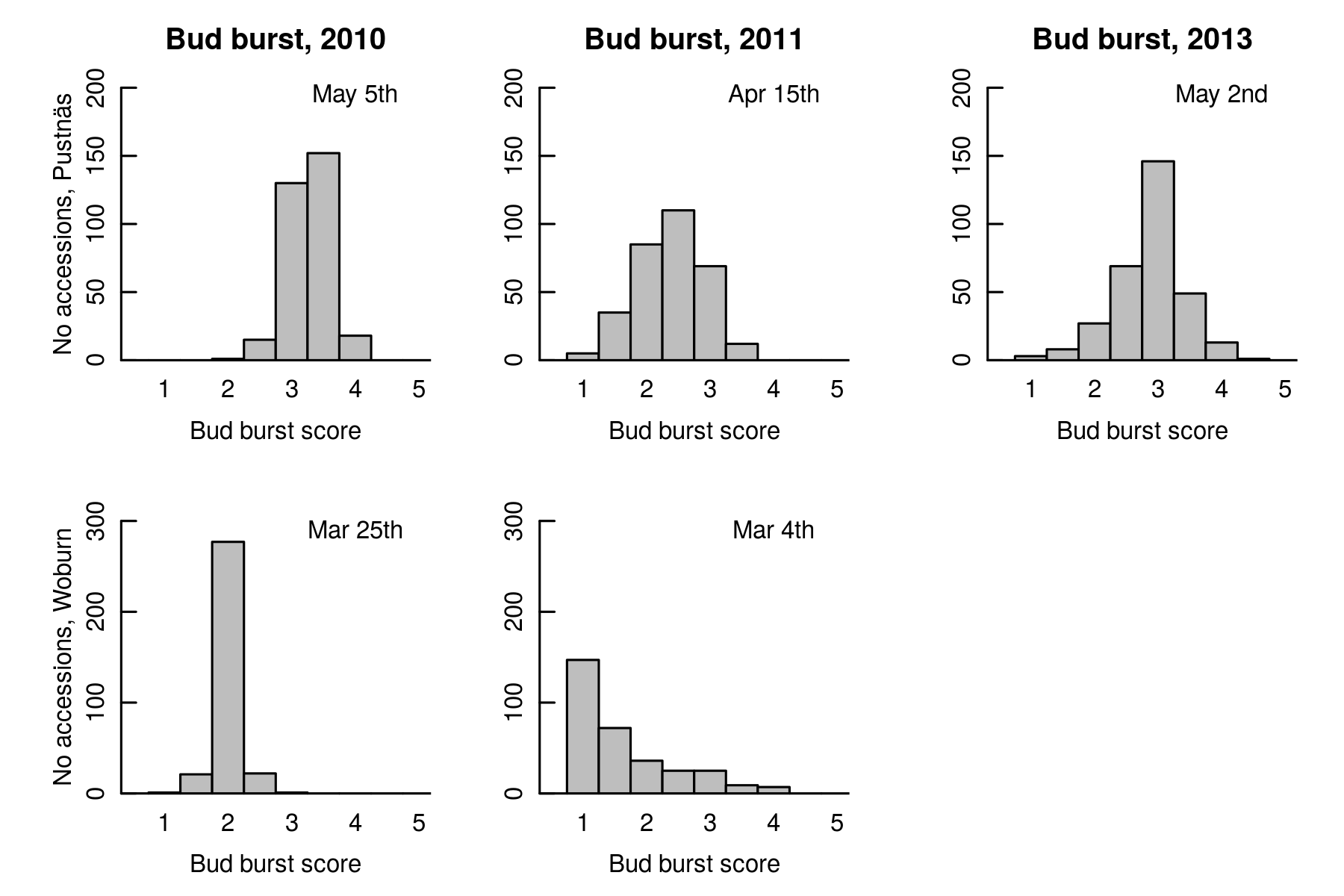

Supplement: Supplementary file 3 — Figure S3. Histogram of the accession estimator distribution for bud burst across years and field trials. [file GCBB-8-670-s003.docx]
