# Supplementary figures and images for: Association mapping in Salix viminalis L. (Salicaceae) – identification of candidate genes associated with growth and phenology
Source: Glob Change Biol Bioenergy. 2015 Jul 29;8(3):670–85. doi: 10.1111/gcbb.12280 (PMC4973673; doi:10.1111/gcbb.12280)

**Fig. S5** : Histogram of the accession estimator distribution for growth cessation in 2010 at Pustnäs.


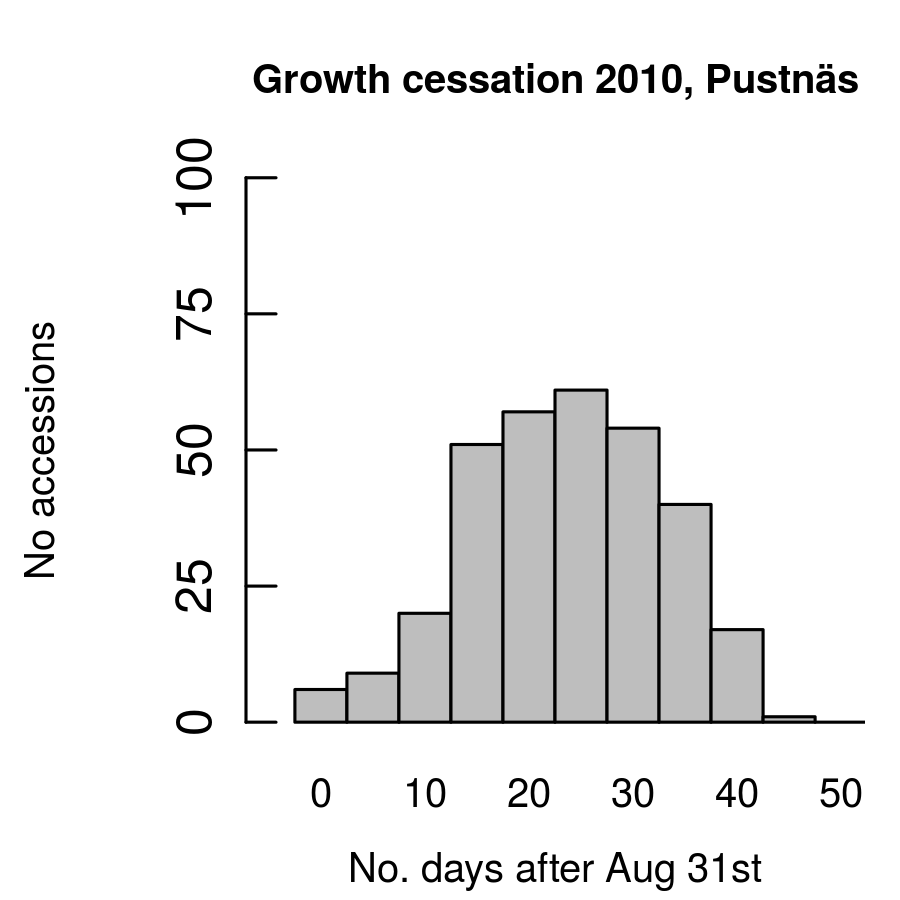

Supplement: Supplementary file 5 — Figure S5. Histogram of the accession estimator distribution for growth cessation in 2010 at Pustnäs. [file GCBB-8-670-s005.docx]
