# Supplementary material for: Association mapping in Salix viminalis L. (Salicaceae) – identification of candidate genes associated with growth and phenology
Source: Glob Change Biol Bioenergy. 2015 Jul 29;8(3):670–85. doi: 10.1111/gcbb.12280 (PMC4973673; doi:10.1111/gcbb.12280)

**Fig. S7** : Probability of finding associations at the false discovery rate 0.2 for all traits in relation to association $R_{adj}^{2}$ (unbiased percentages of variance explained by association)


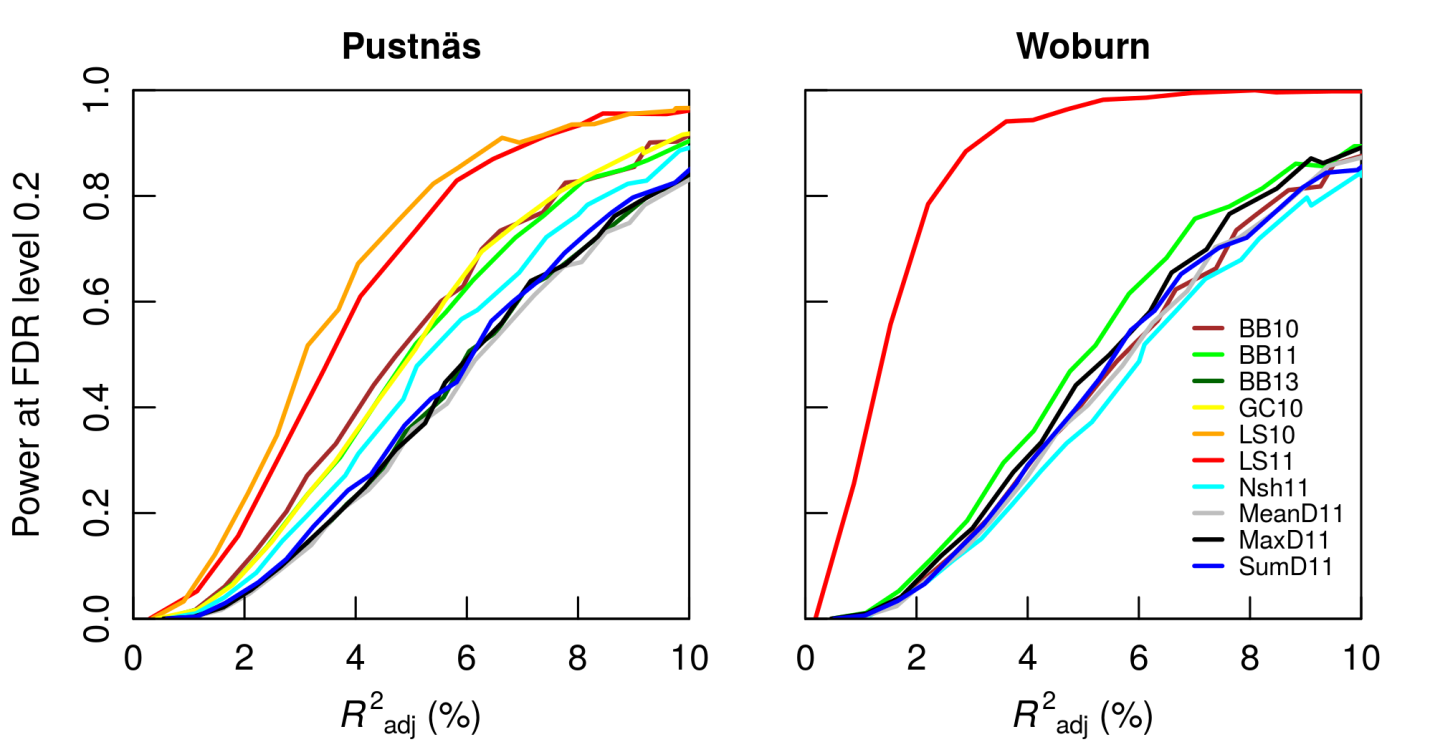

Supplement: Supplementary file 7 — Figure S7. Probability of finding associations at the false discovery rate 0.2 for all traits in relation to association Radj2. [file GCBB-8-670-s007.docx]
