# Supplementary material for: Association mapping in Salix viminalis L. (Salicaceae) – identification of candidate genes associated with growth and phenology
Source: Glob Change Biol Bioenergy. 2015 Jul 29;8(3):670–85. doi: 10.1111/gcbb.12280 (PMC4973673; doi:10.1111/gcbb.12280)

**Fig. S8** : Genotype effects of SNP ZIP1-4494 (a), SBP1-3964 (b) and PtPHYB2-3897 (c) on leaf senescence at both trials. Effects connected with considerable likelihood of true association are marked with: ** *q*<0.01 and *** *q*<0.001. The coloured part of the staples show threshold selection bias adjusted effects while the original biased effect is shown as a black staples in the background. Effects for transformed traits are given in the back-transformed scale and the values in parentheses after each genotype group describe the number of accessions of that group.


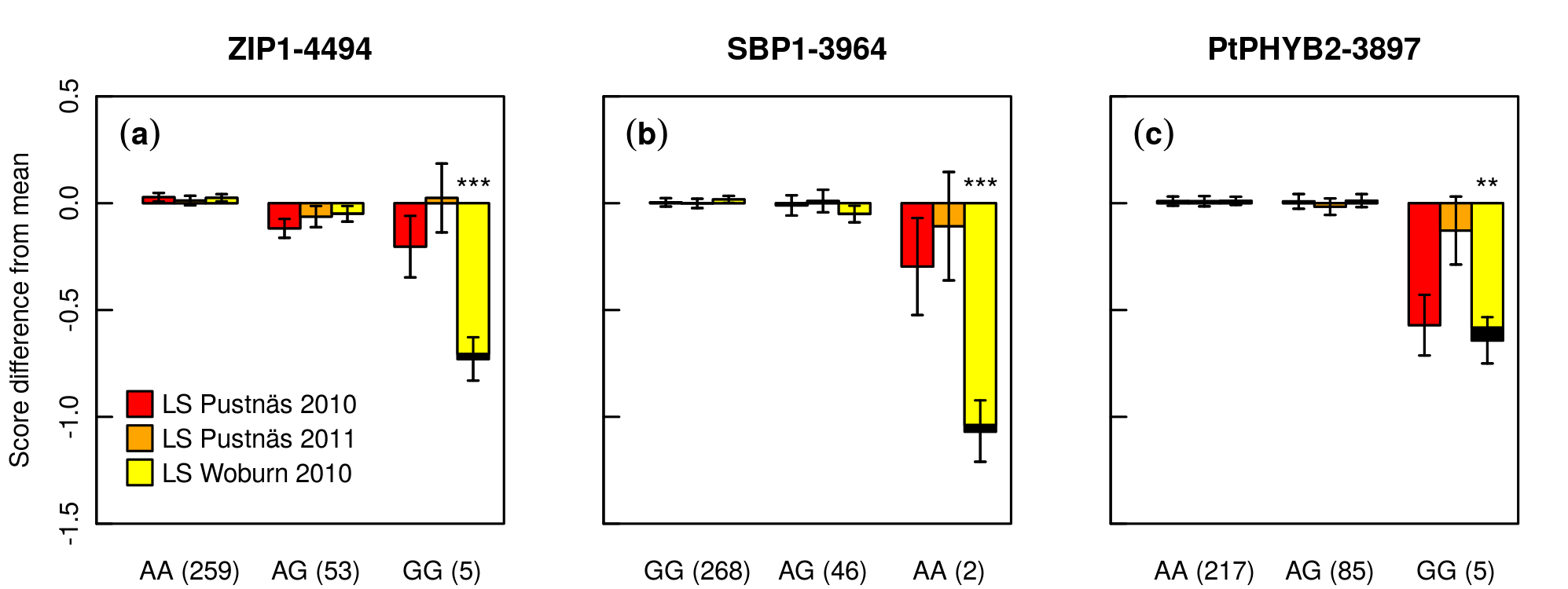

Supplement: Supplementary file 8 — Figure S8. Genotype effects of SNP ZIP1‐4494 (a), SBP1‐3964 (b) and PtPHYB2‐3897 (c) on leaf senescence at both trials. [file GCBB-8-670-s008.docx]
