# Supplementary material for: Association mapping in Salix viminalis L. (Salicaceae) – identification of candidate genes associated with growth and phenology
Source: Glob Change Biol Bioenergy. 2015 Jul 29;8(3):670–85. doi: 10.1111/gcbb.12280 (PMC4973673; doi:10.1111/gcbb.12280)
Supplement: Supplementary file 9 — Supplementary Material and Methods. (A) Salix reference sequence assembly, (B) Significance testing of structure model terms, (C) Multivariate analyses, (D) Adjusting for threshold selection bias by simulation and (E) Derivation of Rps2. [file GCBB-8-670-s009.docx]

Association mapping in *Salix viminalis* L. (Salicaceae) – identification of candidate genes associated with growth and phenology

Henrik R. Hallingbäck^1^*, Johan Fogelqvist^1^, Stephen J. Powers^2^

Juan Turrion-Gomez^3^, Rachel Rossiter^3^, Joanna Amey^3^

Tom Martin^1^, Martin Weih^4^, Niclas Gyllenstrand^1^

Angela Karp^3^, Ulf Lagercrantz^5^, Steven J. Hanley^3^

Sofia Berlin^1^, Ann-Christin Rönnberg-Wästljung^1^

2015-05-20

## Supplementary material and methods

### A. Salix reference sequence assembly

Salix reference sequences for the amplified loci were constructed as follows: First the quality filtered (ConDeTri v2.0, hq=30, minL=70, Smeds & Küstner, 2011) Illumina reads were mapped (Mosaik v1.1 -act 35 -bw 25 -mm 18 -hs 15, Lee *et al.*, 2014) to the poplar reference sequence at the corresponding loci. Duplicated reads were removed (GATK MarkDuplicates v1.104418, McKenna *et al.*, 2010) and variants (SNPs and indels) were subsequently called using Samtools mpileup and vcfutils.pl varFilter, v0.1.12 at default settings (Li *et al.*, 2009). Variants exhibiting allele frequencies above 0.8 across samples were incorporated into the poplar reference whereupon the reads were remapped to this reference. The process was repeated until no new variant could be called. Regions of the processed poplar reference with a high coverage of Illumina reads (>20% median non-zero overage, minimum 90 bp) were retained.

Next a *de novo* assembly was made for each sample, using Velvet v1.04 with K=31 again only using quality filtered reads (ConDeTri v2.0, hq=30, minL=70, Smeds & Küstner, 2011). These *de novo* contigs were then mapped to the poplar reference (including incorporated variants) uding NCBI Blast (v2.2.21, *e*-value cutoff at 10^-5^, Altschul *et al.*, 1990) and the best match for each contig was recorded. For each locus and *Salix* accession, *de novo* sequences with Blast matches and regions of high coverage were assembled with PHRAP (www.phrap.org). The resulting contigs for each locus were aligned (kalign v2.04, default settings, Lassmann & Sonnhammer, 2005) and were subjected to manual inspection/adjustment as deemed necessary. Consensus sequences were thus generated using the most common base at each site and were furthermore compared to known paralogous loci in poplar in order to verify that paralogous loci hadn’t been amplified by mistake. For each locus we also verified that primer sequences used for sequence amplification were consistently present at the ends of the sequence. Finally SNPs were called using the same steps as described for the poplar reference approach.

### B. Significance testing of structure model terms

To address the possibility that either of the **Fq** or **Zu** terms were superfluous, these were subjected to significance testing for each trait omitting the individual SNP term. First, the random term **Zu** was examined by testing log-likelihood ratio between the full (**Fq**+**Zu**) and reduced (**Fq**) models against the $\chi_{df=1}^{2}$ distribution and if non-significant (*p*>0.05) it was thereafter omitted. Subsequently the fixed term **Fq** was subjected to the Wald-F test implemented in ASReml and TASSEL and if non-significant (*p*>0.05) it was omitted. This sequential order of tests was imposed because tests of fixed terms usually assume that random terms are properly treated *a priori* (Welham & Thompson, 1997). For the traits where the tests indicated a reduced model to be preferable (see Table S2), we used that model to redo the association mapping analysis. The results from reduced model analyses were however very similar to those of the full model and for the sake of consistency, only the full model association results are further treated in this study.

### C. Multivariate analyses

In order to formally assess the occurence of SNP associations that were consistent across sites and assessment years (variates), and also SNP associations significantly interacting with sites and years implying G×E-interactions, multivariate forms of the univariate model in eq. 3 were formulated. The multivariate approach taken here is very similar to the multi-trait mixed models initially developed for pedigree based genetic analysis (e.g. Wei & Borralho 1998) but later expanded to accomodate association mapping by Korte *et al.* (2012). As an example, the bivariate form applied for the analysis of accession estimators **y***_es_*_1_ and **y***_es_*_2_ for variates 1 and 2 respectively is shown below:

$\left[ \begin{matrix} \mathbf{y}_{es1} \\ \mathbf{y}_{es2} \end{matrix} \right]=\left[ \begin{matrix} \mathbf{F} & \boldsymbol{0} \\ \boldsymbol{0} & \mathbf{F} \end{matrix} \right]\left[ \begin{matrix} \mathbf{q}_{1} \\ \mathbf{q}_{2} \end{matrix} \right]+\left[ \begin{matrix} \mathbf{S} \\ \mathbf{S} \end{matrix} \right]\mathbf{g}_{c}+\left[ \begin{matrix} \mathbf{S} \\ \boldsymbol{0} \end{matrix} \right]\mathbf{g}_{i}+\left[ \begin{matrix} \mathbf{Z} & \mathbf{0} \\ \mathbf{0} & \mathbf{Z} \end{matrix} \right]\left[ \begin{matrix} \mathbf{u}_{1} \\ \mathbf{u}_{2} \end{matrix} \right]+\left[ \begin{matrix} \mathbf{e}_{es1} \\ \mathbf{e}_{es2} \end{matrix} \right]$ (C1)

Most of the model terms are merely multivariate extensions of eq. 3, but SNP genotype effects were here separated into the **g***_c_*-term which signifies consistent or *common* SNP genotype effects across sites and years (variates), while the **g***_i_*-term signifies SNP genotype effects that *interact* with sites and years. The model is easy to expand further to accomodate more than two variates. All effects were considered to be statistically independent except for the random terms whose variances were assumed to be internally structured as:

$Var\left[ \begin{matrix} \mathbf{u}_{1} \\ \mathbf{u}_{2} \end{matrix} \right]=2\left[ \begin{matrix} \sigma_{A1}^{2} & \sigma_{A12} \\ \sigma_{A12} & \sigma_{A2}^{2} \end{matrix} \right]\otimes\mathbf{K}\mathrm{and}Var\left[ \begin{matrix} \mathbf{e}_{es1} \\ \mathbf{e}_{es2} \end{matrix} \right]=\left[ \begin{matrix} \sigma_{e,es1}^{2} & \sigma_{e,es12} \\ \sigma_{e,es12} & \sigma_{e,es2}^{2} \end{matrix} \right]\otimes\mathbf{I}$ (C2)

where $\sigma_{A1}^{2}$, $\sigma_{A2}^{2}$, $\sigma_{e,es1}^{2}$ and $\sigma_{e,es2}^{2}$ are the additive genetic chip and residual variances for variates 1 and 2 respectively; *σ_A_*_12_ and *σ_e,es_*_12_ are the additive genetic chip and residual covariances between variates 1 and 2; ⊗ is the Kronecker matrix product and **I** is an identity matrix.

Joint multivariate association analyses using this model were performed for all traits that were assessed more than once (several years or sites, Table 1). Thus, bud burst was analysed using a model with five variates, leaf senescence with three variates and for each of the biomass traits (Nsh, MeanD, MaxD, SumD) only two variates. Analyses were then conducted using ASReml (Gilmour *et al.*, 2009) in a manner similar to that of the univariate analyses. However, in similarity to the study of Korte *et al.* (2012) the significance testing for potential SNP-trait associations had to be performed in two separate steps. First, in order to obtain a general unspecific support for SNP-trait associations, Wald-F tests were performed for each SNP and trait for both **g***_c_* and **g***_i_* jointly against the null hypothesis of no association at all (**g***_c_*=**0** and **g***_i_*=**0**). In this scan, the same type of multiple testing correction was applied as for the univariate analyses (Storey & Tibshirani, 2003). In the second step, those SNPs showing a general suggestive/significant association (*FDR-q*<0.2) to a trait were subjected to two additional Walf-F tests. The significance of the common SNP effect (**g***_c_*) was tested in the absence of any interaction SNP effects (setting **g***_i_*=**0**) and subsequently the interaction SNP effect (**g***_i_*) was tested in the presence of **g***_c_*. As the two latter tests are sensitive to variate scale differences, all variates were transformed to a common accession variance by dividing all accession estimators by *σ_c_* prior to multivariate analysis (see eq. 1 and 2). Moreover, as the common and interaction SNP tests only were performed on a subset of SNPs, a multiple testing correction procedure such as that used for the general test was not meaningful. However, a threshold of suggestive significance was still arbitrarily set at *p*<0.001 which is well comparable to the *FDR-q*<0.2 threshold used for many of the other analyses performed in this study.

Apart from testing common and interaction effects of SNP-trait associations *per se*, the overall impact of scale independent G×E-interactions on trait variation was tested by estimating accession correlations between variates adjusted for population structure (Burdon, 1977). This was done by applying the bivariate model shown in eq. C1 to trait pairs (variates) but excluding all terms pertaining to SNP genotypic effects (**g***_c_* and **g***_i_*). Accession variances for each trait 1 and 2 ($\sigma_{s1}^{2}$ and $\sigma_{s2}^{2}$) and covariances between them (*σ_s_*_12_) were then calculated as the sum of the corresponding chip additive and residual (co)variance components respectively (e.g. $\sigma_{s1}^{2}=\sigma_{A1}^{2}+\sigma_{e,es1}^{2}$) and accession correlations were calculated as $r_{s}={\sigma_{s12}}/\left( \sigma_{s1}\sigma_{s2} \right)$.

### D. Adjusting for threshold selection bias by simulation

In order to assess and compensate for the threshold selection bias and to assess the statistical power for the associations, simulated accession estimator data (**y***_si_*) were generated and designed to mimic the presence of artificial SNP effects (**g***_si_*) with a *prespecified* and common percentage of explained variance ($R_{ps}^{2}$). Subsequently this data was subjected to regular univariate association mapping analysis (eq. 3) with the objective of re-estimating the ratio of variance explained ($R_{si}^{2}$) regardless of the prior knowledge. The average *R*^2^-estimate of the significantly associated portions of these simulated data analyses ($\tilde{R}_{si}^{2}$) was then observed to be substantially and systematically larger (overestimated) in comparison to the average -estimate over all simulations ($\overline{R}_{si}^{2}$) which is free from selection threshold bias. Furthermore, because $\tilde{R}_{si}^{2}$ increases with both rising $R_{ps}^{2}$ and $\overline{R}_{si}^{2}$ it was possible to adjust for the selection threshold bias by finding an $\overline{R}_{si}^{2}$ which minimised the difference between the original analysis and simulated analysis ratios of explained variance ($min\left| R^{2}-\tilde{R}_{si}^{2} \right|$, see Allison *et al.*, 2002 and Ingvarsson *et al.*, 2008). Series of simulations were generated for each trait and field trial separately, and for each simulation one of the 1233 investigated SNPs was randomly chosen. Simulated accession estimators were generated as:

$\mathbf{y}_{si}=\mathbf{F}\hat{\mathbf{q}}+\mathbf{S}\mathbf{g}_{si}+\mathbf{Z}\hat{\mathbf{u}}+\mathbf{e}_{si}$ (D1)

where $\hat{\mathbf{q}}$ and $\hat{\mathbf{u}}$ are effect estimates obtained from the ASReml association analysis outputs (eq. 3) of the chosen SNP. Residuals **e***_si_* were randomly drawn from the $N(0,\sigma_{e,es}^{2})$ distribution also using the $\sigma_{e,es}^{2}$ estimate of the original association analysis. To simplify the artificial generation of **g***_si_*, only additive SNP effects were considered
($\mathbf{g}_{si}=\left[ 1 0 -1 \right]^{T}g_{AA}$). SNP effect generation given a specified $R_{ps}^{2}$ could thus be performed by determining *g_AA_* as:

$g_{AA}=\sqrt{\frac{R_{ps}^{2}\sigma_{y-Sg}^{2}}{(1-R_{ps}^{2})(P_{AA}+P_{aa}-\left( P_{AA}-P_{aa} \right)^{2})}}$ (D2)

where $\sigma_{y-Sg}^{2}$ is the estimated variance of the sum of all effects present in eq. D1 except for **Sg***_si_* itself and where *P_AA_* and *P_aa_* are the frequencies of the homozygote genotypes in the sample for the chosen SNP (see also section E). By extensive simulations, Allison *et al.* (2002) showed that in case the assumption of pure additive effects was violated, the method used here may adjust *R*^2^ insufficiently. However the same results also suggested that the remaining threshold selection bias would be minor given that the true effects themselves were small and that adjustments always yielded less biased *R*^2^ than unadjusted estimates even in case SNP effects were dominant/recessive rather than additive.

Subsequently, series of simulated accession predictors were generated for $R_{ps}^{2}$ values in the range 0 to 10% with a resolution of 0.1%. Association mapping analyses using the full model (eq. 3) were performed for these series. Assessment of significance was performed using Wald-F *p* thresholds (*p_th_*) that would closely correspond to the *FDR-q* thresholds applied in the original analysis (*q_th_* at 0.05 or 0.2). Given the relationship between *p* and *q* shown by Storey & Tibshirani (2003), *p_th_* thresholds were calculated for each trait and field trial as:

$p_{th}=\left\{ \begin{aligned} \begin{matrix} q_{th}{\pi_{th}}/{\pi_{0}} & \mathrm{if} \pi_{th}>0 \end{matrix} \\ \begin{matrix} {q_{th}}/{n_{tot}} & \mathrm{if} \pi_{th}=0 \end{matrix} \end{aligned} \right.$ (D3)

where *π_th_* is the proportion of analysed SNPs counted as significantly (or suggestively) associated in the original analysis, *π_0_* is the estimated proportion of true null hypotheses in the original analysis, and *n_tot_* is the total number of SNPs analysed. Using these thresholds it was then possible to select subsets of simulated data analyses in order to manually find the $\overline{R}_{si}^{2}$ that would minimise $\left| R^{2}-\tilde{R}_{si}^{2} \right|$. Such searches were performed for all suggestive or significant associations and the best $\overline{R}_{si}^{2}$-value found for each association was assigned to be the *treshold bias adjusted ratio of variance explained* ($R_{adj}^{2}$). Likewise, as variances and their ratios are based on squares of effects, it was also possible to calculate bias-adjusted SNP effects by using the square root of the adjusted-to-biased quotients: ${\hat{\mathbf{g}}}_{adj}=\frac{R_{adj}}{R}\hat{\mathbf{g}}$. In order to obtain stable and convergent results it was required that the biased $\tilde{R}_{si}^{2}$ estimate was based on a sample of at least 100 $R_{si}^{2}$ estimates of significant associations. Finally, the statistical power for finding SNP-trait associations at *FDR-q*=0.2 was estimated as the proportion of simulations for which *p*<*p_th_* for each trait and potential $R_{adj}^{2}$ estimate (i.e. $\overline{R}_{si}^{2}$).

### E. Derivation of $\boldsymbol{R}_{\boldsymbol{ps}}^{\boldsymbol{2}}$

The prespecified variance ratio of variance explained by an artificial SNP association effect ($R_{ps}^{2}$) can be expanded as:

$R_{ps}^{2}=\frac{\sigma_{ps}^{2}}{\sigma_{ps}^{2}+\sigma_{y-Sg}^{2}}$ (E1)

where $\sigma_{ps}^{2}$ is the variance of the artificial SNP association while $\sigma_{y-Sg}^{2}$ is the variance of $\mathbf{F}\hat{\mathbf{q}}+\mathbf{Z}\hat{\mathbf{u}}+\mathbf{e}_{si}$. The variance of the artificial SNP association is in turn expanded as:

$\sigma_{ps}^{2}=P_{AA}{(g_{AA}-\bar{g})}^{2}+P_{Aa}{(g_{Aa}-\bar{g})}^{2}+P_{aa}{(g_{aa}-\bar{g})}^{2}$ (E2)

where *P_AA_*, *P_Aa_* and *P_aa_* are the frequencies and *g_AA_*, *g_Aa_* and *g_aa_* are the effects of the SNP genotypes *AA*, *Aa* and *aa* respectively, and where $\bar{g}$ is the overall mean effect across genotypes:

$\bar{g}=P_{AA}g_{AA}+P_{Aa}g_{Aa}+P_{aa}g_{aa}$ (E3)

Substituting $\bar{g}$ in eq. E2 with E3 assuming that artificial association effects are strictly additive (*g_aa_*=- *g_AA_* and *g_Aa_*=0) and noting that *P_Aa_*=1- *P_AA_*- *P_aa_*, the expression for $\sigma_{ps}^{2}$ may then be simplified to:

$\sigma_{ps}^{2}=g_{AA}^{2}(P_{AA}+P_{aa}-\left( P_{AA}-P_{aa} \right)^{2})$ (E4)

By solving *g_AA_* out of eq. E4 and $\sigma_{ps}^{2}$ out of eq. E1, the artificial SNP association effects are determined in terms of $R_{ps}^{2}$ and $\sigma_{y-Sg}^{2}$ as:

$g_{AA}=\sqrt{\frac{R_{ps}^{2}\sigma_{y-Sg}^{2}}{(1-R_{ps}^{2})(P_{AA}+P_{aa}-\left( P_{AA}-P_{aa} \right)^{2})}}$

Notably, as this expression is dependent on genotype rather than allele frequencies it does not assume the studied population to conform to Hardy-Weinberg equilibrium.

References

Allison DB, Fernandez JR, Moonseong H, Zhu S, Etzel C, Beasley TM, Amos CI (2002) Bias in Estimates of Quantitative-Trait-Locus Effect in Genome Scans: Demonstration of the Phenomenon and a Method-of-Moments Procedure for Reducing Bias. *American Journal of Human Genetics*, **70**, 575–585.

Altschul SF, Gish W, Miller W, Myers EW, Lipman DJ (1990) Basic Local Alignment Search Tool. *Journal of Molecular Biology*, **215**, 403–410.

Burdon RD (1977) Genetic correlation as a Concept for Studying Genotype-Environment Interaction in Forest Tree Breeding. *Silvae Genetica*, **26**, 168–175.

Gilmour AR, Gogel BJ, Cullis BR, Thompson R (2009) *ASReml User Guide*, VSN International Ltd, Hemel Hempstead, HP1 1ES, UK, 3rd ed.

Ingvarsson PK, Garcia MV, Luquez V, Hall D, Jansson S (2008) Nucleotide Polymorphism and Phenotypic Associations Within and Around the *phytochrome B2* Locus in European Aspen (*Populus tremula*, Salicaceae). *Genetics*, **178**, 2217–2226.

Korte A, Vilhjálmsson BJ, Segura V, Platt A, Long Q, Nordborg M (2012) A mixed-model approach for genome-wide association studies of correlated traits in structured populations. *Nature Genetics*, **44**, 1066–1071.

Lassmann T, Sonnhammer ELL (2005) Kalign – an accurate and fast multiple sequence alignment algorithm. *BMC Bioinformatics*, **6**, 298.

Lee WP, Stromberg MP, Ward A, Stewart C, Garrison EP, Marth GT (2014) MOSAIK: a hash-based algorithm for accurate next-generation sequencing short-read mapping. *PloS One*, **9**, e906581.

Li H, Handsaker B, Wysoker A, *et al.* (2009) The Sequence Alignment/Map format and SAMtools. *Bioinformatics*, **25**, 2078–2079.

McKenna A, Hanna M, Banks E, *et al.* (2010) The genome analysis toolkit: A MapReduce framework for analyzing next-generation DNA sequencing data. *Genome Research*, **20**, 1297–1303.

Smeds L, Küstner A (2011) ConDeTri – A Content Dependent Read Trimmer for Illumina Data. *PLoS One*, **6**, e26314.

Storey JD, Tibshirani R (2003) Statistical significance for genomewide studies. *Proceedings of the National Academy of Sciences of the United States of America*, **100**, 9440–9445.

Wei X, Borralho NMG (1998) Use of individual tree mixed models to account for mortality and selective thinning when estimating base population genetic parameters. *Forest Science*, **44**, 246–253.

Welham SJ, Thompson R (1997) Likelihood Ratio Test for Fixed Model Terms Using Residual Maximum Likelihood. *Journal of the Royal Statistical Society Series B (Methodological)*, **59**, 701–714.
